# The Cell Adhesion Molecules *Roughest*, *Hibris*, *Kin of Irre* and *Sticks and Stones* Are Required for Long Range Spacing of the *Drosophila* Wing Disc Sensory Sensilla

**DOI:** 10.1371/journal.pone.0128490

**Published:** 2015-06-08

**Authors:** Gerit Arne Linneweber, Mathis Winking, Karl-Friedrich Fischbach

**Affiliations:** Department of Neurobiology, Albert-Ludwigs-University Freiburg, Schänzlestr. 1, D-79104, Freiburg, Germany; National Institutes of Health (NIH), UNITED STATES

## Abstract

Most animal tissues and organ systems are comprised of highly ordered arrays of varying cell types. The development of external sensory organs requires complex cell-cell communication in order to give each cell a specific identity and to ensure a regular distributed pattern of the sensory bristles. This involves both long and short range signaling mediated by either diffusible or cell anchored factors. In a variety of processes the heterophilic Irre Cell Recognition Module, consisting of the Neph-like proteins: Roughest, Kin of irre and of the Nephrin-like proteins: Sticks and Stones, Hibris, plays key roles in the recognition events of different cell types throughout development. In the present study these proteins are apically expressed in the adhesive belt of epithelial cells participating in sense organ development in a partially exclusive and asymmetric manner. Using mutant analysis the *GAL4/UAS* system, RNAi and gain of function we found an involvement of all four Irre Cell Recognition Module-proteins in the development of a highly structured array of sensory organs in the wing disc. The proteins secure the regular spacing of sensory organs showing partial redundancy and may function in early lateral inhibition events as well as in cell sorting processes. Comparisons with other systems suggest that the Irre Cell Recognition module is a key organizer of highly repetitive structures.

## Introduction

The perception of the outside world requires highly specialized sense organs, as for example the eyes are for visual stimuli and the ears are for auditory stimuli. The accurate reception of external cues is only possible, if diverse cell types are arranged into complex tissues with the highest precision. In invertebrates, the insect compound eye consists of a regular spaced array of ommatidia forming a biological crystal that allows fast and precise sensing of the environment [1]. In vertebrates, the rows of hair cells in the inner ear are a good example of a well-ordered and repetitive sense organ that allows the tonotopic representation of the auditory world [2]. The development of such precise sensors requires the orchestration of a complex interplay between internal and external signaling events. Especially the latter are even in model organisms still only poorly understood.

The *Drosophila* wing disc is one of the key model organs in the fly and has largely contributed to our understanding of basic developmental concepts like morphogen gradients and compartment boundaries [3]. Furthermore, the fine hairs covering the wings are an important model system to uncover the mechanisms of planar cell polarity [4]. Additionally, although frequently overlooked the *Drosophila* wing is also an important sense organ and arrays of sensory bristles line the anterior edge. The anterior wing margin is comprised of both mechanosensory and chemosensory bristles [5]. The mechanosensory bristles are implicated in flight control [6], while the chemosensory bristles have functions in courtship [7].

The development of any sense organ requires mechanisms to select individual sensory organ precursors (SOPs) to develop into neuronal receptors and adhesion and sorting processes to ensure a regular spaced array. The Notch (N) [8] signaling pathway has been demonstrated in the specification of sense organs in numerous systems including in humans [9]. In the development of sensory bristles in *Drosophila* N inhibits the proneural genes *achaete* and *scute* [10]. Both are expressed in discrete fields with neuronal potential [11]. Initially, all proneural cells express both the receptor *N* and its ligand *Delta* (*Dl*) [12]. Lateral inhibition takes place when spaced nonadjacent cells become strong signaling cells and activate N in the intervening cells. These cells are then inhibited from their neural fate and stop signaling. The importance of N signaling in bristle spacing was demonstrated by a higher density of bristles in hypomorphic *N* and *Dl* mutants [13, 14]. But *N* and *Dl* are not sufficient to maintain spacing over long distances, as both are membrane bound proteins and require direct contact for their function. A protein that has been shown to influence long range spacing is the fibrinogen-related domain protein Scabrous (Sca) [15–17]. Secreted Sca has been shown to be not required for inhibition of neural fate in neighboring cells, but for cells not adjacent to the precursors. Sca functions by changing the adhesive properties of epithelial cells [18].

Cell sorting and adhesive properties of cells are largely influenced by cell adhesion molecules (CAMs), a specialized group of transmembrane proteins that are required throughout development. Four different groups of CAMs have been described: integrins, cadherins, selectins and CAMs of the immunoglobulin (IG) superfamily. The CAMs of the IG group show the highest diversity in structure and possibly also function. The IG proteins Roughest (Rst, also called Irregular chiasm C) [19, 20], Hibris (Hbs) [21, 22], Kin of irre (Kirre, also called Dumbfounded) [23, 24] and Sticks and Stones (SNS) [25] of the Irre cell Recognition Module (IRM) have been described in the development of tissues ranging from the musculature to the central nervous system. In mammals the homologues of the Neph-like proteins Rst and Kirre and of the Nephrin-like proteins Hbs and SNS were originally discovered for their role in podocyte development of the kidney [26, 27]. In more recent years, it has become clear that also the mammalian proteins have important functions in the development of the nervous system [28–32]. All the previous examples, irrespective of the model organism, share that a complex tissue is formed by several highly repetitive subunits through cellular recognition and cell sorting events.

In this paper we explore the roles of the IRM-proteins Rst, Hbs, Kirre and SNS in the spacing of bristles of the anterior wing margin [5, 33–35]. The chemosensory recurved bristles are spaced on average 4.4 cell diameters on the dorsal and 3.8 cell diameters on the ventral side and require therefore long range regulation [5]. We found that the differential adhesion between the IRM-proteins is required for the regular spaced patterns of bristles and that the two groups (Neph- and Nephrin-like proteins) show strong functional redundancy in this tissue.

## Materials and Methods

### *Drosophila* stocks

All stocks were maintained on standard cornmeal molasses agar food at 25°C. Wild type Berlin (*wtb*) was used as wild type stock. The mutant allele *rst* ^*1R34*^ has been described previously [19]. The *GAL4/UAS* technique [36] was used for ectopic gene expression and gene silencing in combination with RNA interference (RNAi) [37]. *MZ1369-GAL4* was used to ubiquitously drive *GAL4* expression in the wing imaginal disc [38], while *neuralized* ^*A101*^ *-GAL4* (*neur-GAL4*) [39], obtained from the Bloomington Stock Center (stock number 6393), was used to specifically drive expression in SOPs. Similarly, *neuralized* ^*A101*^ *-LacZ* (*neur-LacZ*) [40] (Bloomington stock number 4369) was used to mark SOPs. *UAS-rst* [41], *UAS-kirre* [24], *UAS-hbs* [21] and *UAS-sns* [42] where used to ectopically over- or misexpress genes. *UAS-rst-RNAi* and *UAS-hbs-RNAi* (kindly provided by Sujin Bao) [43], *UAS-sns-RNAi*, *UAS-kirre-RNAi* (stock numbers 877 and 27227, VDRC) where used to specifically silence the respective genes. *UAS-mCD8-GFP* [44] was used to outline *GAL4* expressing cells.

### Immunohistochemistry

Immunohistochemistry was performed as previously described [45]. In brief: tissues were dissected and fixed in 4% paraformaldehyde for 20 min on ice. Afterwards they were washed in phosphate buffered saline (PBS) with 0.4% Triton X-100. Antisera were used in following concentrations: anti-LacZ (55976 Cappel/MP; 1:1000), anti-Rst (mAB 24A5; 1:10) [45], anti-Kirre (126i; 1:200) [46], anti-SNS (1:200) [47], anti-Hbs (AS-14; 1:400). The Hbs Antibody AS-14 was produced from a 14 Amino acid peptide AEPSNDDVYSKDDS (1083–1096) by Genscript Corporation using standard protocols. Confocal microscopy of the specimen was carried out with a Leica TCS4D. Comparative experiments were performed using the same laser intensities and by scanning all sets at the same day. Data was processed with AMIRA 5.2 (Indeed, Berlin, Germany) and Adobe Photoshop CS6. All images represent an N number of ≥10.

### Light microscopy

Adult wings where dissected, placed on a microscope slide and where embedded in Canada balsam or glycerol. Photographs were taken using a Zeiss Axioskop2 and a Zeiss Axiocam MRc digital camera. The bristles of the anterior wing margin where counted directly using a low magnification lens under the microscope.

### Statistical analysis

Bristle numbers were analyzed by calculating means and standard error of the mean using Microsoft Excel. Data was tested for normality with the Shapiro-Wilk-test and p-values were calculated with the Students T-test or the Wilcoxon test using R. Recurved bristle distributions were analyzed for each distance in the same manner. Distributions were considered to be significantly different from each other if two distance measurements were statistically different.

## Results

**Fig 1:**
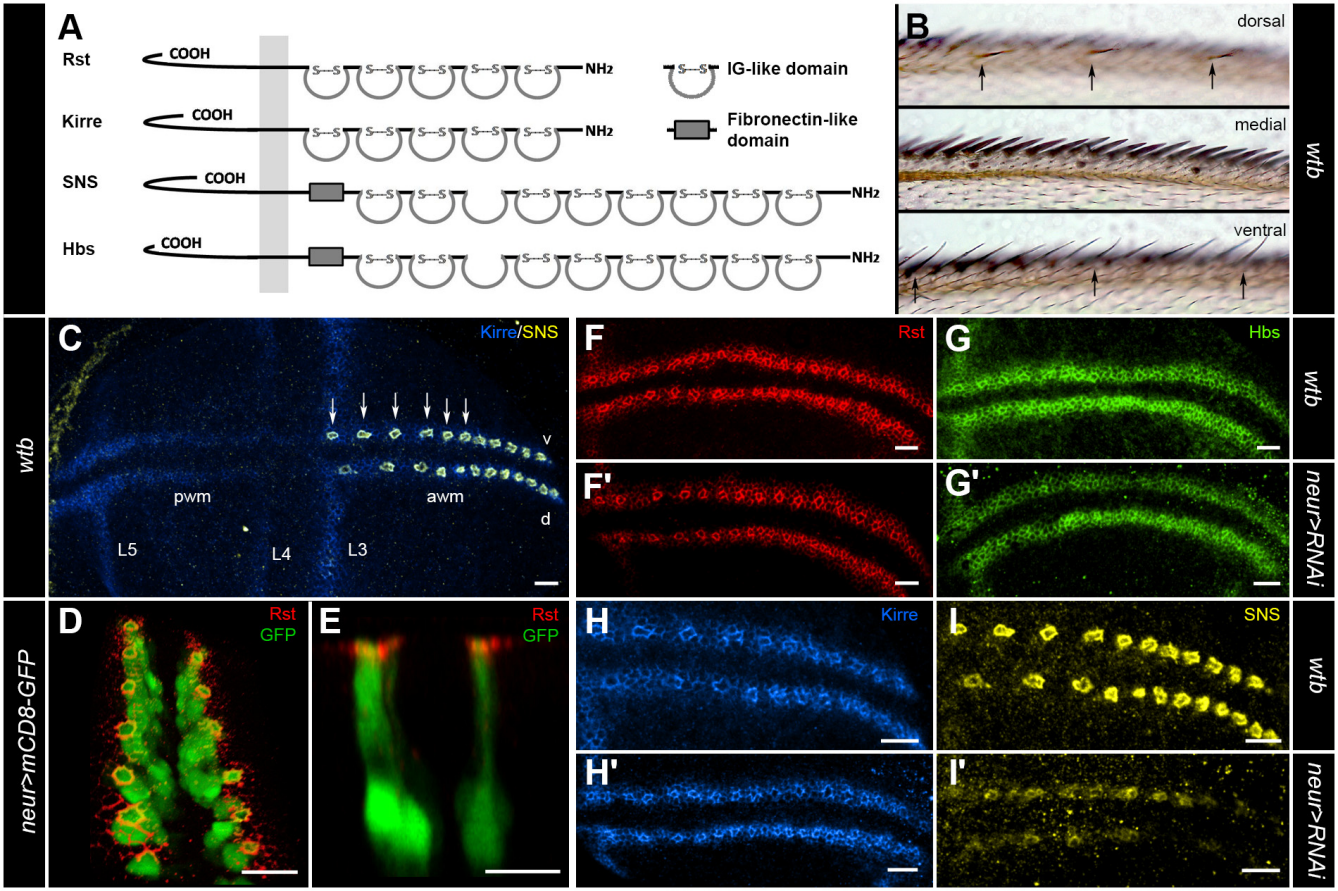
The IRM-proteins Rst, Hbs, Kirre and SNS are expressed in distinct patterns during the development of sensory bristles in the anterior wing margin. (A) The *Drosophila* IRM-proteins consist of the Neph-like proteins Rst and Kirre and of the Nephrin-like proteins Hbs and SNS. (B) Representative image showing the three bristle rows of the anterior wing margin [5]. The dorsal row consists of recurved bristles marked with an arrow. The medial row consists of mechanosensory stout bristles. The ventral row is composed of mechanosensory slender bristles and recurved bristles marked with an arrow. (C) Representative image of the presumptive wing margin of a late third instar larvae. Kirre in blue can be found in the presumptive posterior wing margin (pwm), anterior wing margin (awm) and the wing veins L3, L4 and L5. SNS in yellow can be only found in the SOPs of the awm. Six example SOPs are marked with an arrow. The ventral (v) side faces up and dorsal (d) faces down. (D-E) The apical localization of the IRM-protein Rst (red) is shown in third instar larvae of the genotype *neur-GAL4*>*UAS-mCD8-GFP* (green). The SOPs are specifically marked by GFP. The images show a 3D reconstruction in (D) and a lateral view (E). (F-I) Localization of the IRM-proteins in the awm of third instar larvae. Rst (F) in red is localized in two adhesive belts in the awm and is enriched at the border to the SOPs of the recurved bristles. *neur-GAL4*>*UAS-rst-RNAi* shows no effect on the enrichment of staining around the SOPs (F’). A similar staining pattern in green can be seen for Hbs in wild type (G), but the SOP specific RNAi shows a reduction of staining around the SOP borders (G’), indicating Hbs expression inside the SOPs. Kirre in blue (H) shows a similar pattern as Rst and SOP specific RNAi has no effect (H’). SNS (I) in yellow can only be found at the border of the SOPs. *neur-GAL4*>*UAS-sns-RNAi* reduces SNS in the SOPs (I’). Scale bars correspond to 10μm in all images.

In this paper we show that the IRM-proteins: Rst, Hbs, Kirre, and SNS (Fig 1A) have key functions in the development and spacing of sensory organs in the wing disc of *Drosophila*. We demonstrate that the proteins co-localize in the neurogenic region of the wing margin, and that they have a strong regulatory effect on each other. The dominant function of these two highly redundant sets of Neph- and Nephrin-like proteins [34] is to secure a regular spaced array of sensory bristles (Fig 1B) via cell recognition and sorting. Loss of function or misexpression of the IRM-proteins lead to phenotypes ranging from a disruption of the chemosensory recurved bristle spacing to fully missing or malformed bristles.

### The IRM-proteins in the anterior wing margin

Strong IRM-protein immunoreactivity and gene expression (by RNA *in situ* hybridization, data not shown) were detected in the presumptive wing margin of the larval wing disc. In the third larval instar all four IRM-proteins were detected in the neurogenic region of the presumptive anterior wing margin and in the wing veins (Fig 1C–1I). The neurogenic region consists of two cell types: SOPs and SOP surrounding cells. The IRM-proteins mediated contact between these cell types on the apical side in the adherens junction (Fig 1D–1E). The expression patterns of Rst, Kirre and Hbs looked almost identical (Fig 1F–1H). All three proteins were found at the cell borders of all cells in the neurogenic region and were enriched around the SOPs that give rise to the recurved bristles. Nevertheless, the cellular localization of Rst and Kirre was different from Hbs. Tissue specific RNAi in the SOPs showed no reduction of the SOP margin immunoreactivity for Rst and Kirre (Fig 1F’–1G’, indicating that neither Rst or Kirre are expressed in SOPs), but it did for Hbs (Fig 1H’, indicating that Hbs is indeed expressed in SOPs. SNS was in contrast to the other three IRM-proteins only detected in the membranes surrounding the SOPs and was similar to Hbs reduced by tissue specific RNAi in the SOPs (Fig 1I–1I’). In summary, all four IRM-proteins were expressed in distinct patterns throughout the development of the sensory bristles of the anterior wing margin.

### The Neph-like proteins Rst and Kirre act redundantly to secure the spacing of the recurved bristles

**Fig 2:**
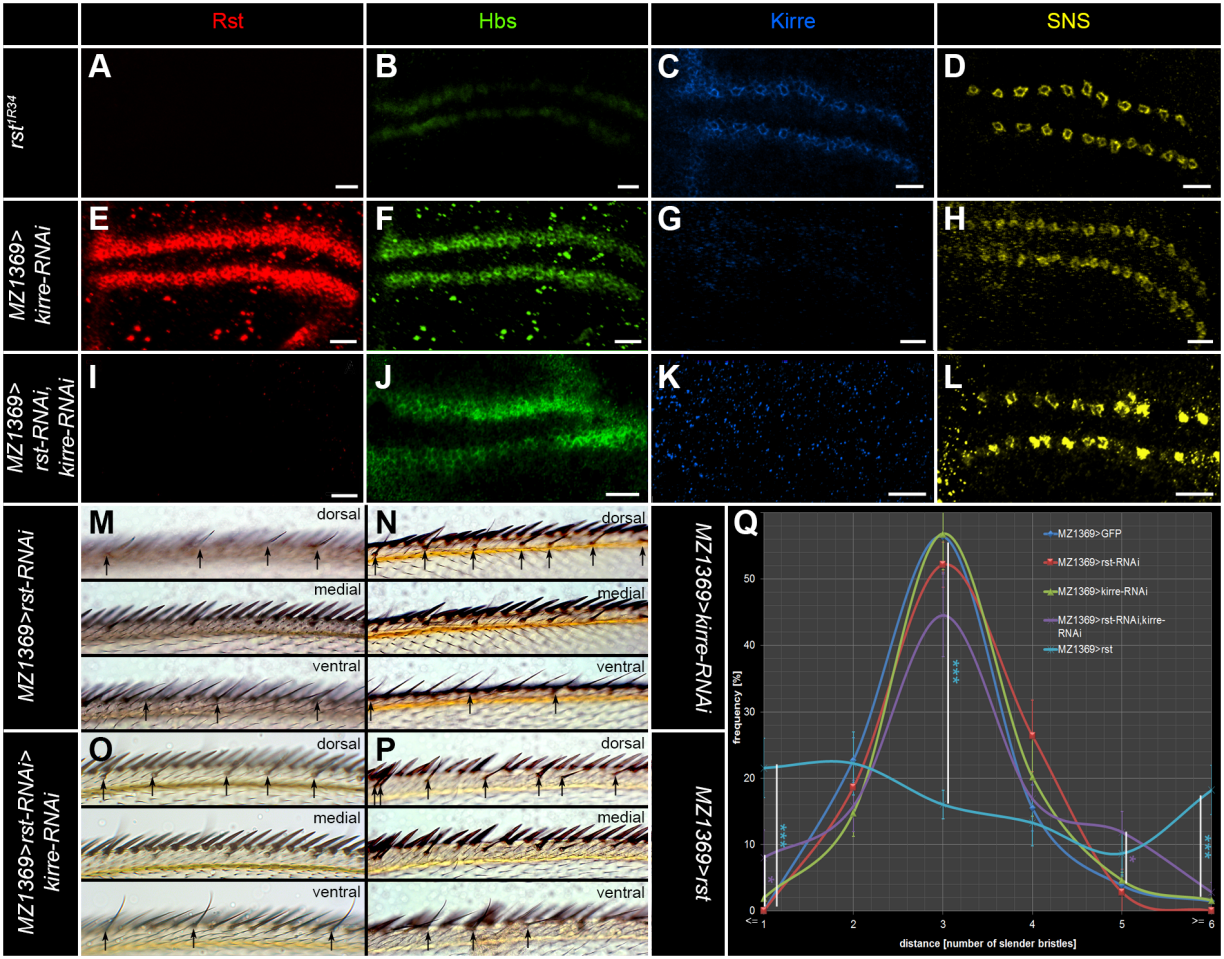
Rst acts cooperatively with Kirre to secure the bristle pattern in the anterior wing margin. (A-L) Projection views of IRM-protein immunoreactivity in late third instar larvae. Rst is shown in red (A, E, and I), Hbs in green (B, F and J), Kirre in blue (C, G and K) and SNS in yellow (D, H and L). (A-D) The *rst* allele *rst* ^*1R34*^ shows no detectable Rst staining (A). (B) Hbs staining is reduced in the membranes surrounding the SOPs and is mainly detected in SOP membranes. Kirre (C) and SNS (D) show no significant pattern change. (E-H) *MZ1369-GAL4*>*UAS-kirre-RNAi* shows no significant changes of the Rst (E) and Hbs pattern (F). Kirre immunoreactivity is hardly detectable (G) while SNS (H) is mildly reduced. (I-L) In the *rst*, *kirre* double RNAi hardly any Rst (I) and Kirre (K) can be detected. Enrichment of Hbs (J) around SOPs is reduced and the SOP arrangement as seen with SNS (L) is severely disrupted. (M) In the adult *MZ1369>rst-RNAi* shows a mild spacing phenotype with spacing ranging from 2 to 5 intervening bristles. (N) *MZ1369>kirre-RNAi* shows a mild spacing phenotype with spacing ranging from 1 to 7 (similar data was obtained for the mutant *rst* ^*1R34*^, data not shown). (O) *MZ1369>rst-RNAi*, *kirre-RNAi* shows a significant disturbance of the spacing of recurved bristles with 0 to 8 intervening cells. (P) *MZ1369-GAL4* misexpression of *rst* has a strong impact on the spacing of recurved bristles with spacing ranging from 0 to 13 intervening cells. Clustered recurved bristles are frequently observed and similarly long areas without any chemosensory bristles are seen. (Q) shows the quantitative analysis of the recurved bristle spacing as measured by the number of slender bristles between the recurved bristles. The distribution of *MZ1369>GFP* differs significantly from *MZ1369>rst-RNAi*, *kirre-RNAi* in the following spacing values: < = 1: p-value = 0.034, 5: p-value = 0.041. *MZ1369>rst* differs significantly in the following spacing values: < = 1: p-value = 0.0002, 3: p-value = 0.0002, > = 6: p-value = 0.0001. Scale bars correspond to 10μm in all images.

The similarity of expression patterns for Rst and Kirre suggested that the two proteins could act redundantly. In agreement with this idea, the analysis of single gene loss of function wing discs, for *rst in the* mutant *rst* ^*1R34*^ [19] (Fig 2A–2D, S1A–S1D Fig and by RNAi, data not shown) and *kirre* by RNAi (Fig 2E–2H and S1E–S1H Fig), showed no severe disruption of the SOP pattern. Noteworthy, we observed a mild down-regulation of the preferred partner in both cases. The preferred interaction partner is Hbs for Rst [43] and SNS for Kirre [48]. The *rst* and *kirre* double knockdown showed in contrast to the single gene manipulations a clear disruption of the SOP pattern, including wing margin areas without any SOPs and SOP clusters (Fig 2I–2L and S1I–S1L Fig). In the adult wing margin, the knockdown of single IRM genes caused in agreement with the larval data only mild spacing defects (Fig 2M, 2N and 2Q). No significant changes in bristle numbers were observed (Table 1). The double knockdown (Fig 2O and 2Q) resulted in much more severe defects, strongly affecting the bristle spacing. This data was similar to *rst* ^*vt*^ an allele that affects both *rst* and *kirre* expression [34]. Even more severe effects on bristle spacing were observed by overexpressing one of the Neph-like proteins (*rst*, Fig 2P and 2Q and *kirre*, S3Q, S3R and S3U Fig). These manipulations lead to almost randomly distributed recurved bristles. Additionally, we observed misplacements of the other sensory bristles (slender, stout) into small groups of 3 to 5 and we observed changes in the numbers of sensory organs (Table 1). Our data shows that the Neph-like proteins Rst and Kirre serve redundant functions in the spacing of sensory sensilla in the wing disc.

**Table 1:** Bristle numbers on dorsal and ventral side of the anterior wing margin.

genotype		dTr	mTr	mTr/dTr	vTr r	vTr s	vTr s/vTr r
***wtb***	**Mean**	**18.1**	**83.6**	**4.6**	**12.4**	**45.5**	**3.7**
(n = 10)	SE	± 0.4	± 1.3	± 0.1	± 0.3	± 1.2	± 0.1
***MZ1369>mCD8-GFP***	**Mean**	**19.6**	**82.9**	**4.2**	**14.4**	**45.7**	**3.2**
(n = 10)	SE	± 0.4	± 1.6	± 0.1	± 0.4	± 1.2	± 0.1
***rst^1R34^***	**Mean**	**17.6**	**81.0**	**4.7**	**12.6**	**46.9**	**3.8**
(n = 10)	SE	± 0.6	± 1.1	± 0.2	± 0.6	± 1.1	± 0.2
***MZ1369>rst-RNAi***	**Mean**	**18.5**	**87.8**	4.8 *	**15.0**	**48.9**	**3.3**
(n = 10)	SE	± 0.6	± 2.0	± 0.2	± 0.4	± 0.9	± 0.1
***MZ1369>rst***	**Mean**	15.4 ***	**81.7**	5.4 **	**11.9**	41.7 *	**3.8**
(n = 10)	SE	± 0.7	± 2.4	± 0.3	± 1.0	± 1.2	± 0.5
***MZ1369>kirre-RNAi***	**Mean**	**18.6**	**80.8**	**4.4**	**14.8**	**48.3**	**3.3**
(n = 10)	SE	± 0.6	± 2.7	± 0.1	± 0.6	± 1.4	± 0.1
***MZ1369>kirre***	**Mean**	14.5 ***	69.7 *	4.9 *	13.0 *	36.4 **	2.8 *
(n = 10)	SE	± 0.8	± 3.1	± 0.2	± 0.5	± 1.3	± 0.1
***MZ1369>rst-RNAi*, *kirre-RNAi***	**Mean**	**20.2**	**84.0**	**4.2**	**14.5**	**49.4**	**3.5**
(n = 10)	SE	± 0.5	± 3.2	± 0.2	± 0.7	± 1.0	± 0.1
***MZ1369-GAL4>UAS-hbs-RNAi***	**Mean**	**18.7**	**88.5**	4.8 *	**14.1**	**48.5**	3.5 *
(n = 10)	SE	± 0.5	± 1.9	± 0.1	± 0.4	± 1.0	± 0.1
***MZ1369-GAL4>UAS-hbs***	**Mean**	14.5 ***	**81.1**	5.8 **	**12.5**	40.8 *	**3.4**
(n = 10)	SE	± 0.8	± 2.0	± 0.4	± 0.8	± 1.3	± 0.2
***MZ1369-GAL4>UAS-sns-RNAi***	**Mean**	**20.2**	**79.7**	**4.0**	**14.6**	**46.2**	**3.2**
(n = 10)	SE	± 0.6	± 1.8	± 0.1	± 0.3	± 0.6	± 0.1
***MZ1369-GAL4>UAS-sns***	**Mean**	**20.2**	**84.9**	**4.2**	**13.9**	**44.6**	**3.3**
(n = 10)	SE	± 0.7	± 1.9	± 0.2	± 0.8	± 1.1	± 0.1
***MZ1369>hbs-RNAi*, *sns-RNAi***	**Mean**	**19.1**	**82.0**	**4.3**	**15.2**	**45.6**	**3.0**
(n = 10)	SE	± 0.5	± 2.3	± 0.1	± 0.8	± 1.2	± 0.1

The table shows the mean and standard error (SE) of the bristle counts from several genotypes used in this study. The first column shows the genotypes. The data columns are named as follows: dorsal triple row (dTR), middle triple row (mTr), dTR/mTr, ventral triple row recurved bristles (vTr r), ventral triple row slender bristles (vTr s) and vTr r/vTr s. Mean numbers shown are always half male half female as no sex differences were observed. Values that differ significantly from the controls are marked with an asterisk (*) (T-test < 0.05) and with two asterisks for (T-test < 0.01) and three for (T-test <0.001).

### The Nephrin-like proteins Hbs and SNS act to a certain extent redundantly to secure the spacing of the recurved bristles

**Fig 3:**
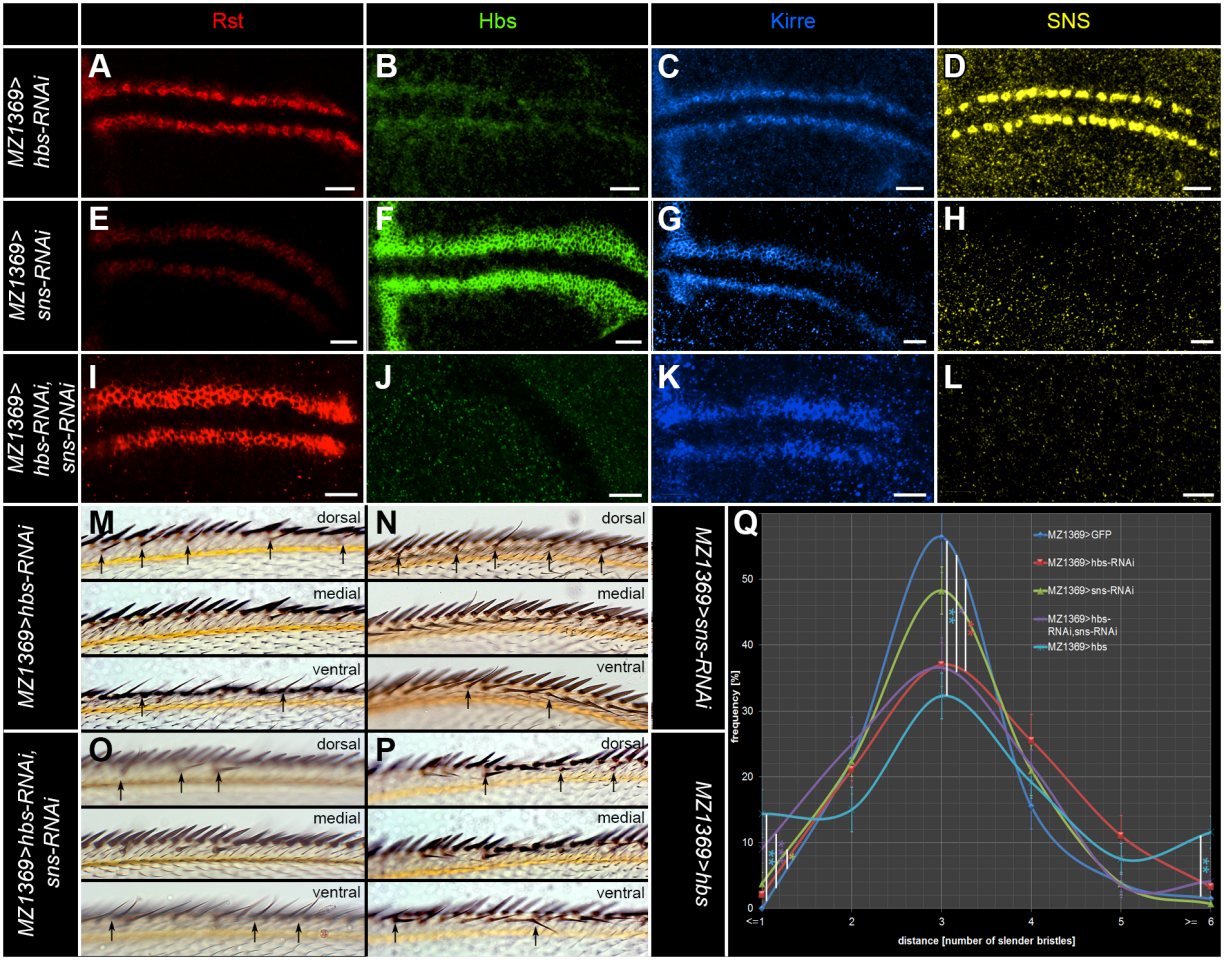
Hbs acts cooperatively with SNS to secure the bristle pattern in the anterior wing margin. (A-L) Projection views of IRM-protein immunoreactivity in late third instar larvae. Rst is shown in red (A, E and I), Hbs in green (B, F and N), Kirre in blue (C, G and K) and SNS in yellow (D, H and L). (A-D) Global *hbs-RNAi* using *MZ1369-GAL4* reduces the staining for Rst (A) and Kirre (C) in all membranes that are not in contact to the SOPs (B) Hbs immunoreactivity is reduced and no clear membrane localization is detectable. (D) SNS immunoreactivity is only mildly affected, but SOPs stand significantly nearer to each other. (E-H) *MZ1369-GAL4*>*UAS-sns-RNAi* shows mildly reduced Rst staining (E). Hbs (F) and Kirre (K) immunoreactivity is unchanged. SNS (H) is not detectable. (I-L) In the double RNAi *MZ1369>hbs-RNAi*, *SNS-RNAi* only the two adhesive belts with Rst (I) and Kirre (K) are visible, but no obvious SOPs are marked. Hbs (J) and SNS (L) are not detectable. (M) In the adult *MZ1369-GAL4* driven *hbs-RNAi* shows only a mild spacing phenotype with 0 to 7 intervening cells. (N) The global *sns-RNAi* in the entire wing disc shows a very mild spacing phenotype with spacing ranging from 1 to 5. (O) *MZ1369>hbs-RNAi*, *sns-RNAi* shows a significant disturbance of the spacing of recurved bristles with spacing ranging from 0 to 8. (P) *MZ1369-GAL4* driven misexpression of *hbs* has a strong impact on the spacing of recurved bristles with spacing ranging from 0 to 12. Additionally, clustered recurved bristles are frequently observed. (Q) Quantitative analysis of the recurved bristle spacing, as measured by the number of slender bristles between the recurved bristles. The distribution of *MZ1369>GFP* differs significantly from *MZ1369>hbs-RNAi* in the following spacing value: 3: p-value = 0.009. *MZ1369>SNS-RNAi* differs significantly in the following spacing value: < = 1: p-value = 0.035. The double RNAi for *hbs* and *SNS* is significantly different for: < = 1: p-value = 0.0002, 3: p-value = 0.014. *hbs* overexpression differs for: < = 1: p-value = 0.0011, 3: p-value = 0.0013, > = 6: p-value = <0.001. Scale bars correspond to 10μm in all images.

The single gene RNAi knockdowns of *hbs* (Fig 3A–3D and S2A–S2D Fig) and *sns* (Fig 3E–3H, S2E–S2H and S4B Figs for independently labelled SOPs) caused only a mild effect on the spacing of the SOPs. Only in the double knockdown of both genes was the SOP pattern of the neurogenic region significantly disrupted (Fig 3I–3L, S2I–S2L and S4C Figs). The functional redundancy of Hbs and SNS is surprising, as the proteins showed radically different expression patterns in the wing margin. In the adult wing, we observed similar phenotypes to the larval ones. We found a mild disruption in the single knockdowns (Fig 3M–3N and 3Q), while the double knockdown and the overexpression of the Nephrin-like proteins showed severe disruptions of the bristle pattern (Fig 3O–3Q and S3S–S3U Fig). Additionally, we saw in the *hbs* misexpression that mechanosensory bristle numbers were significantly increased, resulting in a change of average spacing of chemosensory bristles (Table 1). Furthermore, we observed frequent disruptions of wing veins (data not shown). In summary, our data shows that the Nephrin-like proteins SNS and Hbs have partially redundant functions in the spacing of sensory sensilla in the wing margin.

### Targeted misexpression of Neph-like proteins shows preferential adhesion and interactions in *cis* and *trans*

**Fig 4:**
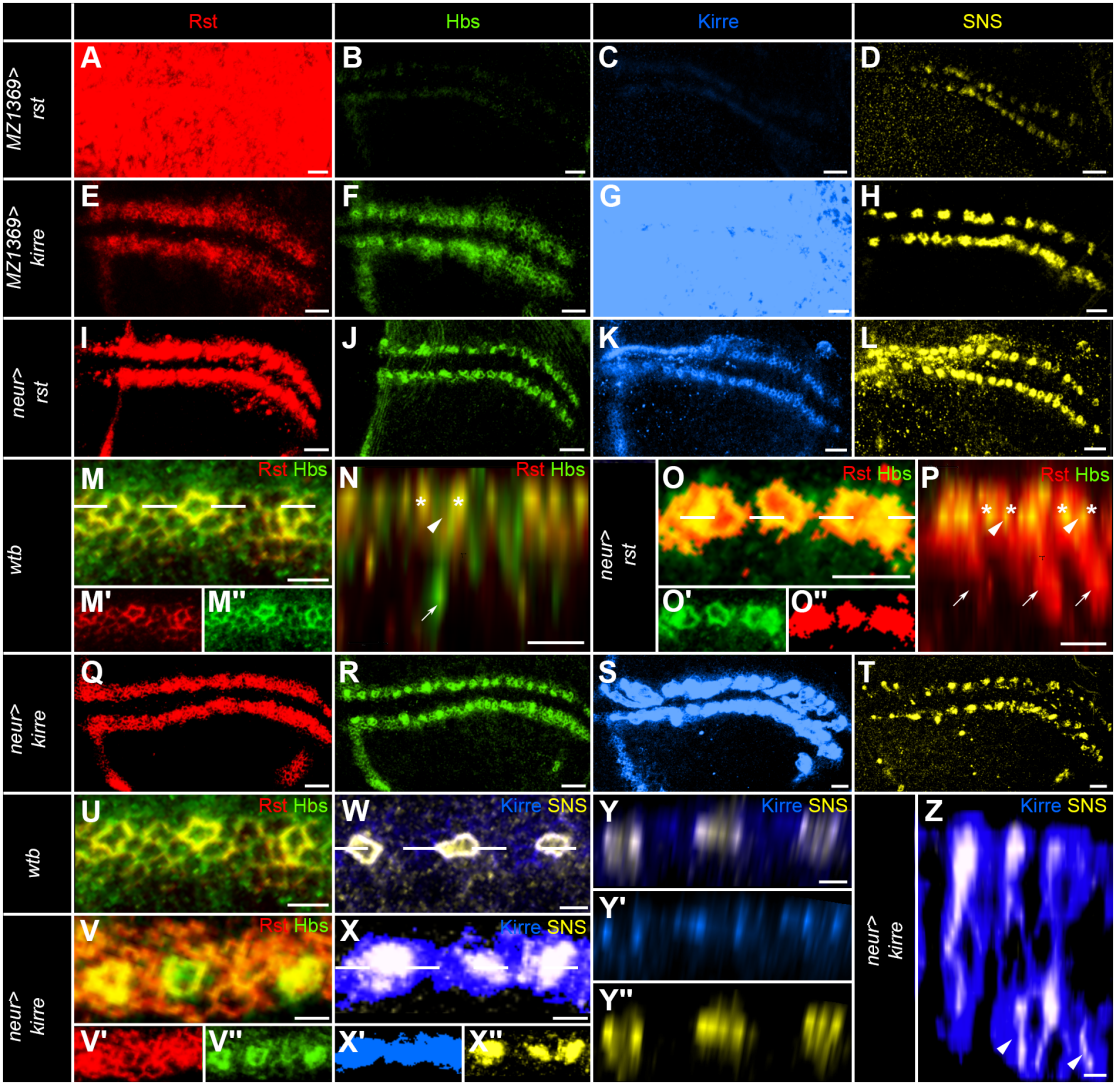
The Neph-like proteins Rst and Kirre affect the localization of other IRM members in *cis* and *trans*. (A-M, O, Q-X) Projection views of IRM immunoreactivity in third instar larvae. Rst is shown in red (A, E, I, M-Q and U-V), Hbs in green (B, F, J, M-P, R and U-V), Kirre in blue (C, G, K, S and W-Z) and SNS in yellow (D, H, L, T and W-Z). (A-D) Misexpression of *rst* using *MZ1369-GAL4* leads to ubiquitous Rst staining (A) in the entire wing disc. Hbs (B) and Kirre (C) are significantly reduced. SNS (D) staining is unaffected in strength, but the localization is not limited to the apical contact zone of the SOPs. Instead it is found in the entire cell. Additionally, the order of the SOPs is severely disturbed. (E-H) Misexpression of *kirre* via *MZ1369-GAL4* leads to wider stripes of Rst (E) and Hbs (F) staining. Kirre (G) can be ubiquitously detected in the entire wing disc. SNS (H) staining is strong on all membranes in contact with Kirre positive membranes. Spacing of SOPs is already disrupted at this developmental stage. (I-L) Misexpression of *rst* using *neur-GAL4* leads to strongly stained Rst (I) positive SOPs. Hbs (J) is found only around the SOPs and staining of membranes not in contact to the SOPs is reduced. (K) Kirre staining is further enriched around the SOPs. (L) SNS is relocated and it is not specifically located at the adherens junction any more. (M) Magnification of three SOPs of a wild type control stained for Rst and Hbs. The dashed line shows the approximate area of cut for the Z-projection in (N). Hbs can be detected inside the SOPs (arrowhead) and also in the basal appendix (arrow). (O) Magnification of three SOPs in *neur-GAL4*>*UAS-rst*. The approximate area of cut for the Z-projection in (P) is given with a dashed line. Arrowheads mark the immunopositive interior of the SOPs showing strong Rst staining. Arrows marks the basal appendix of the SOPs. Asterisk mark the co-localization of Rst and Hbs immunoreactivity at the Border of SOPs. (Q-T) Misexpression of *kirre* using *neur-GAL4* leads to strong Rst staining around the SOPs. Hbs (R) is found much stronger around or in the SOPs and is strongly reduced on the membranes not in contact with any SOPs. (S) Kirre staining is strongly found in all membranes of the SOPs, showing no apical-basal polarity. (T) SNS localization inside the SOPs is disrupted and a possible degradation product can be found in vesicles basal of the adherens junction. (U) Magnification of three SOPs of a wild type control stained for Rst and Hbs. (V) Magnification of three SOPs in *neur-GAL4*>*UAS-kirre*. Strong accumulation of Hbs immunoreactivity is evident around the SOPs. (W) Magnification of three SOPs of a wild type control stained for Kirre and SNS. (X) The magnification of three SOPs of *neur-GAL4*>*UAS-kirre* shows the strong Kirre staining in the entire SOP and the mislocalization of SNS. A dotted line in (W and X) shows the approximate area of a Z-cut that is shown in (Y and Z). In wild type (Y) both proteins interact with each other only in a defined apical contact zone. The distribution of the proteins is clearly visible. SNS is inside the SOPs and Kirre in the surrounding cells. In (Z) the distribution of Kirre and SNS is shown in the mutant situation. Mislocalization and vesicular degradation products of SNS can be seen basal of the adherens junction in this z-axis view. Scale bars correspond to 10μm in all images.

To explore the *in vivo* interactions of the IRM-proteins, we made use of ubiquitous and cell-type-specific misexpression experiments. Ubiquitous misexpression of *rst* had strong effects on the localization of all IRM-proteins (Fig 4A–4D and S3A–S3D Fig). Rst was detected in the entire wing disc and apical-basal localization was lost. Interestingly, overexpression of *rst* lead to strongly reduced signals for Hbs and Kirre. SNS positive SOPs were severely disrupted and SNS immunoreactivity was no longer located primarily in the adherens junctions of the SOPs, but it was instead more broadly distributed in the SOPs. Ubiquitous *kirre* misexpression on the other hand (Fig 4E–4H and S3E–S3H Fig) increased the width of Rst and Hbs positive adhesive belts and many SOPs, as indicated by SNS staining, were directly touching each other. Both misexpressions of the Neph-like proteins resulted in strong adult bristle spacing phenotypes (Fig 3P and 3Q and S3Q–S3R and S3U Fig) and in changes of bristle numbers (Table 1).

Targeted misexpression of *rst* in the SOPs had compared to the ubiquitous misexpression very different effects on Hbs and Kirre (Fig 4I–4P and S5A–S5D Fig). Hbs was strongly enriched around the borders of the SOPs and reduced in cell membranes not in contact to SOPs. Similar was true for Kirre, which could be found predominately around the SOPs as well. SNS was displaced inside the SOPs in a similar manner to the global misexpression and did not localize primarily in the adherens junction anymore. Z-axis cuts through the SOPs showed in comparison to the wild type control no Hbs inside the SOPs but Rst instead (Fig 4M–4P). In the adult wing, the SOP specific misexpression had severe effects on bristle spacing and bristle numbers (S5Q, S5U Fig and S1 Table).

Similar effects were observed, when misexpressing *kirre* in the SOPs using *neur-GAL4* (Fig 4Q–4Z and S5E–S5H Fig). Almost identical to the SOP specific *rst* misexpression, Rst and Hbs immunoreactivity strongly accumulated around the SOPs, but neither Rst nor Hbs (Fig 4Q, 4R and 4V) were removed entirely from the membranes not in contact to the SOPs. Z-axis cuts through the SOPs showed that Kirre was mislocated to the entire SOP membrane and that the SOP cell appendixes cluster. Furthermore, SNS was found in basally located vesicles (Fig 4W–4Z). In the adult, severe bristle defects were observed similar to the *rst* misexpression (S5R, S5U Fig and S1 Table).

### Targeted misexpression of Nephrin-like proteins shows changes in cell shape and strong preferential adhesion with Neph-like proteins in *trans*

**Fig 5:**
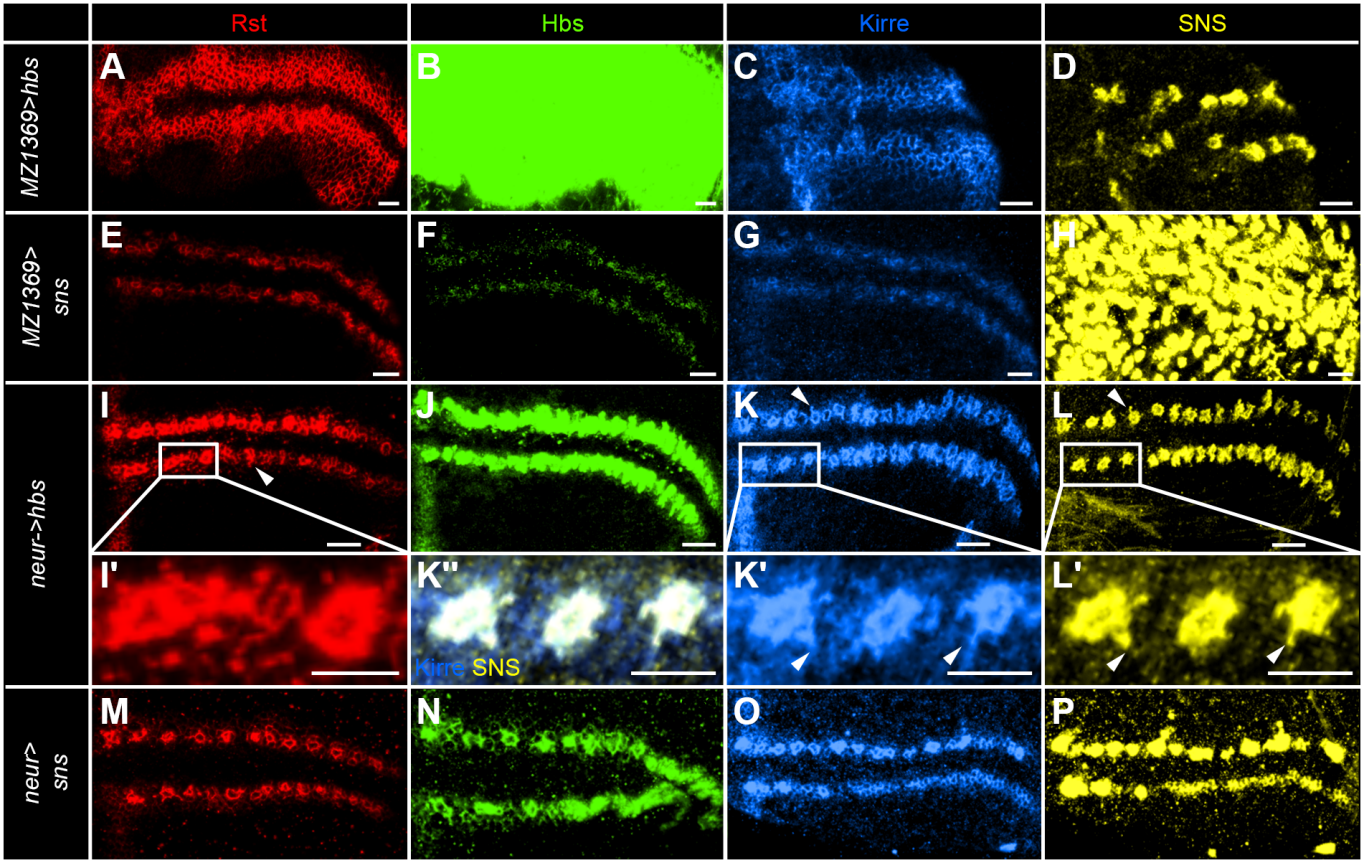
The Nephrin-like proteins Hbs and SNS affect other IRM-proteins asymmetrically and induce filopodial extensions. (A-L) Projection view of IRM immunoreactivity in late third instar larvae. Rst is shown in red (A, E, I and M), Hbs in green (B, F, J and N), Kirre in blue (C, G, K and O) and SNS in yellow (D, H, L and P). (A-D) Misexpression of *hbs* using *MZ1369-GAL4* leads to a significant enlargement of Rst (A) and Kirre (B) immunoreactivity positive areas. Specific enrichment of immunoreactivity around the SOPs is lost. Hbs (C) is ubiquitously located in all membranes. SNS (D) staining shows that the SOPs have lost their regular pattern already in this developmental stage. (E-H) Ubiquitous misexpression of *sns* via *MZ1369-GAL4* leads to reduced staining of Rst (E), Hbs (F) and Kirre (G). SNS (H) staining shows patched expression. (I-L) Overexpression of *hbs* via *neur-GAL4* leads to strongly Rst stained membranes in contact with the SOPs (I). Membranes not in contact to the SOPs show reduced staining. This is also evident in an enlargement of the Rst staining (I’). (J) Hbs staining can be found in the entire SOPs without profound apical basal polarity. (K) Kirre is similar to Rst enriched at the membranes in contact to the SOPs. Hardly any staining can be found in other membranes of the adhesive belt. (L) Cellular localization of SNS is only mildly affected by the *hbs* misexpression as the protein is mainly, but not exclusively localized at the SOP membrane. Additionally, filopodial outgrowths are frequently observed in this genotype as marked with arrowheads in (I), (K) and (L) and in the enlargements (K’) and (L’). (M-P) Overexpression of *sns* using *neur-GAL4* leads to strongly enriched staining of Rst (M), Hbs (N) and Kirre (O) around the SOPs. (P) SNS can be found along all membranes of the SOPs due to the overexpression. Similar to hbs overexpression are filopodial outgrowth frequently observed. Scale bars correspond to 10μm in all images.

Ubiquitous misexpression of *hbs* increased the width of the Rst and Kirre positive adhesive belts (Fig 5A–5D and S3I–S3L Fig). SNS positive SOPs were almost randomly localized in the neurogenic adhesive belts and their numbers were reduced. In the adult wing, fewer recurved bristles were found and these were not evenly distributed along the wing margin any more (S3S, S3U Fig and Table 1). Ubiquitous misexpression of *sns* resulted in slim and size reduced adhesive belts as visualized by Rst, Hbs and Kirre antibodies (Fig 5E–5H and S3M–S3P Fig). Increased immunoreactivity around SOPs was not visible with any IRM-protein and instead cells with increased signals were found in unsorted clusters. These were located in some cases several cell diameters away from the presumptive neurogenic region. SOP specific labelling with *neur-LacZ* shows that the SOPs pattern is indeed strongly disorganized and several SOPs are displaced away from the neurogenic band (S4D Fig). In the adult, *sns* misexpression had a strong effect on the spacing of sensory organs (S3T–S3U Fig and Table 1)

Overexpression of *hbs* in the SOPs strongly attracted Rst and Kirre to the Border of the SOPs (Fig 5I–5L and S5I–S5L Fig). The effect was even stronger for Rst than Kirre. Additionally to changing the protein localization across the membrane, we did observe increased filopodial outgrowths of the SOPs (Fig 5I’–5L’). In the adult, *hbs* overexpression in SOPs had only mild effects on bristle spacing, but significantly increased their numbers (S5S, S5U Fig and S1 Table). Similarly to *hbs*, the overexpression of *sns* in the SOPs affected the adhesive properties of the cells only mildly (Fig 5M–5P and S5M–S5P Fig). Rst, Hbs and Kirre strongly accumulated in the cell membranes in contact with the SOPs. In this particular case Kirre accumulation was stronger than Rst. SNS was localized on the entire SOPs membrane losing its apical-basal localization and we observed similar to *hbs* overexpression an increase in filopodial outgrowths. In the adult, overexpression of *sns* affected the spacing of bristles only mildly (S5T–S5U Fig and S1 Table).

The data presented here shows that the IRM-proteins Rst, Hbs, Kirre and SNS act cooperatively to secure the regular spaced array of sensory bristles in the anterior wing margin. Both the Neph-like proteins Rst, Kirre and the Nephrin-like proteins Hbs and SNS showed functional redundancy. Interestingly, we could show by changing the composition of IRM-proteins in the wing disc that the proteins have strong regulatory effects on the distribution of each other *in cis* and *in trans*.

## Discussion

The IRM-proteins Rst, Hbs, Kirre and SNS have been shown to be involved in a number of developmental processes e.g. axonal pathfinding, eye development, muscle fusion and antennal disc development [19, 20, 22, 23, 25, 38, 41, 43, 45]. Similar statements can also be made for the mammalian homologues the Neph and Nephrin proteins [26–32]. In most *Drosophila* organs—with maybe the exception of muscle fusion—the function of each single protein is presently not cleared up. This present paper presented evidence that all four IRM-proteins are involved in the spacing of the chemosensory bristles serving partly redundant functions. This redundancy has been previously found for Rst and Kirre and Hbs and SNS in a variety of other systems [23, 48].

### Differential gene expression and polarity of the IRM-proteins in the wing disc

In epithelia, CAMs form complexes in the adherens junctions, giving the proteins an apical-basal localization [49]. The IRM-proteins in the presumptive wing margin showed not only apical-basal polarity, but also differential gene expression between different cell types and polarized localization of protein in the adjacent cell boundaries of the SOPs of the recurved bristles. This differential gene expression and polarized localization of proteins resembles partly the situation of cell sorting in the eye disc [34, 41, 43].

**Fig 6:**
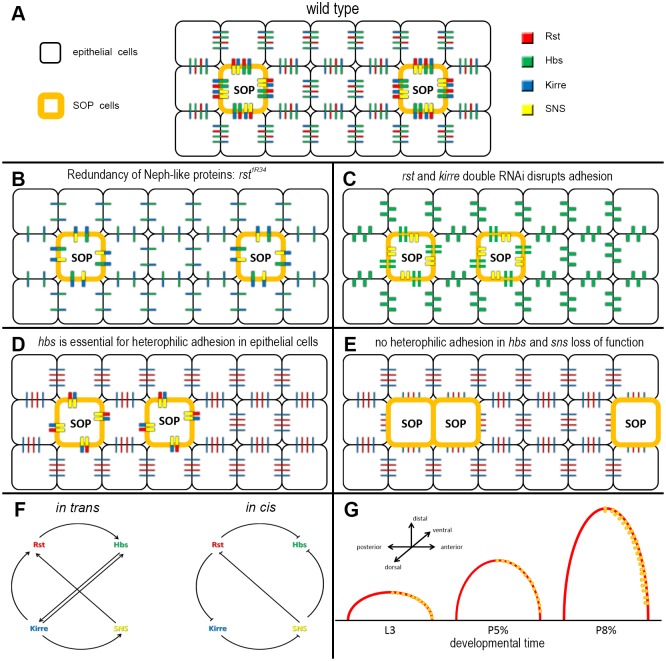
Model of IRM-protein interactions in the *Drosophila* anterior wing margin. (A-E) Illustration of IRM-protein functions in the anterior wing margin. In black are epithelial cells shown, while SOPs are shown in orange. Protein interactions are shown in different sizes according to the strength of the interaction. Red represents Rst, green Hbs, blue Kirre and SNS is shown in yellow. In the wild type (A) preferred adhesion was observed between the SOPs and the surrounding epithelial cells. Epithelial cells are additionally stable connected through the Hbs, Rst and Hbs, Kirre interaction. In *rst* ^*1R34*^ (B), as an example for Neph-like loss of function, preferential adhesion can still be observed between the SOPs and epithelial cells through the SNS, Kirre and Hbs Kirre interaction. Only in the case of Rst and Kirre loss, the adhesive properties of the wing margin is changed leading to bristle clusters (C). Loss of Hbs prevents heterophilic interaction between the non-SOP cells resulting in mild disturbances of the SOP pattern (D). Loss of Hbs and SNS results in total loss of heterophilic interaction between all cell types in the presumptive anterior wing margin (E). This results in strong disturbances of the SOP and later the bristle pattern (F) Summary of the inductive and competitive interactions between the IRM-proteins *in trans* and *in cis*. In the interaction between two cells *in trans* several inductive events were observed, if these events represent inductions of gene expression or stabilization of proteins in the adhesive belt by heterophilic interactions is currently unknown. Inside cells *in cis* several competitive interactions were observed, resulting in degradation of proteins in vesicles. Altogether, these interactions allow a precise regulation of IRM-protein abundance and function. (G) Chain model of preferential adhesion of IRM-proteins in the wing disc. The IRM-proteins in the wing disc secure a strong adhesive chain in the distal growing wing. Preferential adhesion around the SOPs secures a constant high number of cells between the SOPs. Growth in distal directions explains the lower number of cells between precursors compared to the adult sensory organs.

In wild type (summarized in Fig 6A), Rst and Kirre are located in the membranes of the SOP surrounding cells. However, they are strongly enriched at the membranes adjacent to a SOP, similarly to the protein distribution of Rst in interommatidial cells of the eye disc [41, 43]. Hbs is expressed in the surrounding cells and in the SOPs, where its main function may be to stabilize Rst and Kirre *in trans* in opposite cells and to regulate the effect of SNS. SNS is only found in the SOPs. This differential gene expression is the reason for the strong enrichment of Rst and Kirre immunoreactivity around the SOPs. The asymmetric distribution of Rst and Kirre around the SOPs can be understood as a form of planar cell polarity [4], giving each cell an orientation in a single plane. The aim of this localized distribution is to space the sensory bristles and to stabilize them in their position. This model is based on the differential gene expression of IRM-proteins after the segregation of neuronal precursors by lateral inhibition in the antennal disc [38] and additionally based on the findings of the eye disc [41, 43] and movement of precursors in the wing disc [18, 50].

### IRM-proteins are required for the spacing of sensory organs in the wing disc

Removal of one of the Neph-like proteins (Rst or Kirre) has little effect on the adhesive properties in the presumptive wing margin as they are largely redundant in this system and they can compensate each other (Fig 6B). Only if both Neph-like proteins are removed (Fig 6C), all specific interaction between SOP and non-SOP cells are lost, as SNS and Hbs are not able to interact homophilic [42]. This leads to freely moving SOPs that are not kept at their specific position.

Similar to the removal of a single Neph-like protein does the removal of a single Nephrin-like protein (Hbs or SNS) only mildly interfere with the IRM interactions in the wing disc. As long as one Nephrin-like protein is expressed in the SOPs, they are stabilized by the strong interaction with the Neph-like proteins (Fig 6D). In the case of SNS, only the expression in the SOPs is lost and this can be compensated by the Hbs expression in SOPs. Hbs is additionally required to stabilize Rst and Kirre in the cells around the SOPs. In the case of Hbs loss, the interaction between non-SOP cells can be compensated by Rst and Kirre that can weakly interact homophilic [21, 45]. In the case both Nephrin-like proteins are lost the SOPs lose all specific adhesion (Fig 6E). In this situation only the adhesive belt shows hemophilic interaction between the Neph-like proteins.

Ubiquitous misexpression of all IRM-proteins caused diverse changes in the stability of the IRM resulting in severe disruptions of the SOP and bristle positions. More interestingly are the SOP specific mis- or overexpressions that showed little or no effect with the Nephrin-like proteins Hbs and SNS and drastic effects like bristle and SOP clustering with the Neph-like proteins Rst and Kirre. This clustering of bristles can be understood by the homophilic adhesion of Neph-like proteins Rst and Kirre [21, 45]. They can be found in these experiments in high concentrations around the cell borders of the bristle precursors leading to strong adhesive forces between adjoined cells. This results in the clustered phenotype similar to the fasciculation of nerve fibers in the optic lobe [19, 20, 45].

### The impact of IRM-proteins on the number of sensory organs

Additionally to the observed cell spacing phenotypes, especially misexpression of IRM-proteins did alter the numbers of sensory organs in the wing disc, indicating a possible regulatory role in N signaling [51]. This is true for both the ubiquitous and cell-type-specific misexpression experiments. As the ubiquitous misexpression experiments change protein distributions in different cell types, they are difficult to interpret. More meaningful are the cell-type-specific misexpression experiments of IRMs in the SOPs. In these experiments it was crucial whether a Neph-like or Nephrin-like protein was misexpressed or overexpressed. In case of *Rst* and *kirre* misexpression the number of recurved bristles was dramatically reduced. *sns* overexpression reduced the numbers only slightly, while *hbs* increased their number. An explanation for the increase of sensory bristles lies in the requirement for *hbs* in N signaling [52]. In this model hbs overexpression would lead to increased N signaling allowing the specification of more bristles. The reductions of sensory bristles resulting from *rst* or *kirre* misexpression could indicate either a direct negative regulatory effect of these proteins on N signaling or this could be explained by their effects on Hbs levels in the SOPs. The opposite effects of SNS and Hbs on bristle number is similar to the antagonistic modulation seen in muscle fusion [21] and could indicate a negative regulatory role for SNS on N signaling. In summary, this data suggests that the IRM-proteins are important for the specification and identity of the SOPs in development. Failure of having the correct protein configuration leads to developmental defects.

### IRM-proteins regulate levels and the localization of each other

One of the striking features of the IRM-proteins are their abilities to alter the protein localization and the size of the expression domains of the others. While the former may be explained by direct protein-protein interactions, the latter could be explained by protein degradation or hint to a transcriptional regulatory network as indicated in the eye [53]. The regulative interactions of IRM-proteins are summarized in Fig 6F.

#### Rst affects Hbs and Kirre protein levels

The *rst* loss of function mutant *rst* ^*1R34*^ showed a decrease of Hbs immunoreactivity. This is especially striking as it was until recently [54] not possible to show adhesion between Rst and Hbs *in vitro* [21, 42]. Nevertheless, *in vivo* heterophilic extracellular interaction between the two proteins was shown to play an important role in the eye disc [43]. The reduction of the Hbs protein in the wing disc upon loss of Rst could be caused by the absence of stabilizing heterophilic interactions *in trans*. In the opposite situation, when *rst* was ubiquitously misexpressed, Hbs as well as Kirre protein levels were reduced. This might be explainable by protein degradation due to *cis*-interactions along the membrane [43]or could be related to transcriptional regulation [53].

### Kirre stabilizes SNS and Hbs *in trans* and Kirre degrades SNS *in cis*

The phenotypic effects of *kirre* loss and gain of function resembles largely the observations for *rst*. Nevertheless we observed also distinct differences. Loss of *kirre* reduces significantly SNS protein levels on the SOP membrane showing the requirement for Kirre to stabilize SNS *in trans*. Interestingly, ubiquitous misexpression did not strongly affect Rst or Hbs localization or levels, while *kirre* misexpression *in cis* in the SOPs leads to vesicular degradation. This *cis*-interaction gives evidence for the importance of IRM-proteins in cellular identity.

### Hbs organizes the IRM competence in the wing margin

Hbs is the only IRM-protein that is not differentially expressed in the development of the wing margin. The protein could be detected inside the SOPs and in the surrounding cells. In the loss of function situation for *hbs*, the neurogenic zones, as measured by IRM-protein expression, became significantly smaller. The opposite was observed when *hbs* was ubiquitously overexpressed and significantly wider stripes were measured. This differential behavior in the two experiments allows the conclusion that Hbs has an important regulatory effect on the other IRM-proteins in SOP surrounding cells. But again it remains unknown, whether this can be explained via stabilization of proteins in the membrane or due to transcriptional regulation. If this is indeed due to transcriptional regulation, N regulation is highly likely [52]. Inside the SOPs Hbs attracts the Neph-like IRM-proteins to the opposite membrane, serving an important function in the SOP/epithelial interaction which to some degree resembles the situation found in the eye [43].

#### SNS attracts the other IRM-proteins *in trans* and represses them *in cis*

The main function of SNS is to attract the other IRM-proteins *in trans*, which can be increased by overexpressing *sns* in the SOPs. The strong repression of other IRM-proteins in the *sns* misexpression experiment underlines the importance of IRM-proteins in cellular identity.

### The requirement for the IRM-proteins in bristle spacing

The perception of the world requires highly precise sense organs. The wing margin bristles are by no means an exception to this rule [5]. The development of such sensors requires a complex temporal and spatial interplay of genes and their corresponding proteins. The process starts with the induction of proneural genes by wingless signaling along the presumptive wing margin [55, 56]. The wingless positive stripe is surrounded by two discrete fields with neuronal potential [11]. In this fields lateral inhibition by N signaling regulates the number of SOPs and induces an initial spacing [12–14]. Hbs has been implicated to play a role in N signaling and γ-secretase processing [52]. However our RNAi experiments have not uncovered any major changes in bristle numbers in the wing margin. Either our drivers where expressed too late, the knockdown was incomplete or this specific Hbs function can be compensated in the wing margin by another protein, possibly SNS. On the other hand, our over- and misexpression experiments did indicate a strong and tightly regulated role for the IRM-proteins in cellular identity and cell differentiation. However, N signaling and lateral inhibition are insufficient to explain the approximately 4 to 5 cell diameter spaced bristles [5]. Instead we propose that the regular long range spaced bristles are a result of the movements resulting from the invagination of the wing pouch and of strong chain-like adhesion between SOP and non-SOP cells in the wing margin (Fig 6G). Filiopodial outgrowths of the bristle precursors presumably increase their range and prevent clustering [50]. A key factor regulating cellular adhesion in bristle development is Sca [18, 50], a protein that has direct regulatory functions for Rst [38]. In the chain model we have the strongest heterophillic adhesion between SOPs (Hbs, SNS) and non-SOP cells (Rst, Kirre, see also Fig 6A). Weaker heterophillic adhesion is found in non-SOP cells through Rst, Kirre and Hbs. The adhesive force generated by these differential protein distributions keep bristle precursor regular spaced and allow only few cells to enter the adhesive belt while the wing pouch is expanding. This complex process is likely mediated by other CAMs as well [57], but heterophillic adhesion by IRM-proteins seems to be crucially required. Furthermore, apoptosis mediated cell death could further refine the process [58].

### IRM-proteins as organizers of highly repetitive structures

The involvement of IRM-proteins have been shown in a number of developmental contexts [20, 23, 43, 45, 59, 60]. All these developmental systems share a common feature with the bristles of the wing disc, as they are all highly repetitive structures. In most of these systems the distributions and the specific functions of the individual IRM-protein differs from one system to the next, but the task of the module to ensure regular patterned tissues shows interesting similarities. For example, SNS is essential for muscle fusion, but only minor important in eye development [25], vice versa is true for Hbs [21, 22, 43]. The IRM triggers in both cases recognition between different cell types. In brain development *rst* loss and gain of function leads to fasciculation of neurons. This effect is similar to the clustering of sensilla seen in the wing disc and also in the antennal disc [38]. From this point of view fasciculation can also be understood as a failure of spacing. This suggests that this module of proteins is a widely used developmental organizer of repetitive structures that has been individually fine-tuned during evolution for different organs.

## Supporting Information

S1 Fig (A-L) High magnification images of projection views of IRM-protein immunoreactivity in late third instar larvae. Rst is shown in red (A, E, and I), Hbs in green (B, F and J), Kirre in blue (C, G and K) and SNS in yellow (D, H and L). (A-D) The *rst* allele *rst* ^*1R34*^ shows no detectable Rst staining. (B) Hbs staining is reduced, especially in the membranes surrounding the SOPs and the protein is mainly detected in SOP membranes. Kirre (C) and SNS (D) show no significant pattern change. (E-H) *MZ1369-GAL4*>*UAS-kirre-RNAi* shows no significant changes of the Rst (E) and Hbs pattern (F). Kirre immunoreactivity is hardly detectable (G) while SNS (H) is mildly reduced. (I-L) In the *rst*, *kirre* double RNAi hardly any Rst and Kirre (I, K) can be detected. Enrichment of Hbs (J) around SOPs is reduced. SNS (L) is not evenly distributed around the SOP membrane. Scale bars correspond to 10μm in all images.
(TIF)

S2 Fig (A-L) High magnification images of projection views of IRM-protein immunoreactivity in late third instar larvae. Rst is shown in red (A, E and I), Hbs in green (B, F and N), Kirre in blue (C, G and K) and SNS in yellow (D, H and L). (A-D) Global *hbs-RNAi* using *MZ1369-GAL4* reduces the staining for Rst (A) and Kirre (C) in all membranes that are not in contact to the SOPs (B) Hbs immunoreactivity is reduced and no clear membrane localization is detectable. (D) SNS immunoreactivity is mildly stronger. (E-H) *MZ1369-GAL4*>*UAS-sns-RNAi* shows mildly reduced Rst staining. Hbs (F) and Kirre (K) immunoreactivity is unchanged. SNS (H) is not detectable. (I-L) In the double RNAi *MZ1369>hbs-RNAi*, *SNS-RNAi* the two adhesive belts with Rst (I) and Kirre (K) are visible, but no obvious SOPs. Hbs (J) and SNS (L) are not detectable. Scale bars correspond to 10μm in all images.
(TIF)

S3 Fig (A-P) High magnification images of projection views of IRM immunoreactivity in third instar larvae. Rst is shown in red (A, E, I and M), Hbs in green (B, F, J and N), Kirre in blue (C, G, K and O) and SNS in yellow (D, H, L and P). (A-D) Misexpression of *rst* using *MZ1369-GAL4* leads to ubiquitous Rst staining (A). Hbs (B) and Kirre (C) are significantly reduced and enrichment around SOPs is not visible anymore. SNS (D) staining is unaffected in strength, but the localization is not limited to the apical contact zone of the SOPs. Instead it is found in the entire cell. (E-H) Misexpression of *kirre* via *MZ1369-GAL4* leads to wider and less membrane specific stripes of Rst (E) and Hbs (F) staining. Kirre can be ubiquitously detected (G). SNS (H) staining is strong on all membranes in contact with Kirre membranes. (I-L) Misexpression of *hbs* using *MZ1369-GAL4* leads to a significant enlargement of Rst (I) and Kirre (L) immunoreactivity positive areas. All membranes of the adhesive belt show increased immunoreactivity and specific enrichment around the SOPs is lost. Hbs (K) is ubiquitously located in all membranes. SNS (L) staining shows that the SOPs have lost their regular pattern already in this developmental stage. (M-P) Ubiquitous misexpression of *sns* via *MZ1369-GAL4* leads to reduced staining of Rst (M), Hbs (N) and Kirre (O). SNS (P) staining shows patched expression. (Q) In the adult *MZ1369-GAL4* misexpression of *rst* has a strong impact on the spacing of recurved bristles and spacing ranges from 0 to 13 intervening cells. Clustered recurved bristles are frequently observed and also long areas without any chemosensory bristles are seen. (R) *MZ1369-GAL4* driven *kirre* misexpression has a strong impact on the spacing of recurved bristles with spacing ranging from 0 to 7. Clustered recurved bristles are frequently observed similarly are also long are areas without any chemosensory bristles seen. (S) *MZ1369-GAL4* misexpression of *hbs* has a strong impact on the spacing of recurved bristles with values ranging from 0 to 8. Clustered recurved bristles are frequently observed. (T) *MZ1369-GAL4* driven *sns* misexpression impacts on the spacing of recurved bristles with spacing ranging from 0 to 5. Quantitative analysis of the ventral spacing can be seen in (U). The distribution of *MZ1369>GFP* differs significantly from *MZ1369>rst* in the following spacing value: < = 1: p-value = 0.0002, 3: p-value = 0.0002, > = 6: p-value = 0.0001. *MZ1369>kirre* differs significantly in the following spacing value: < = 1: p-value = 0.0002, 3: p-value = 0.0002. *MZ1369>hbs* differs significantly in the following spacing value: < = 1: p-value = 0.0012, 3: p-value = 0.0013, > = 6: p-value = 0.001. *MZ1369>sns* differs significantly in the following spacing value: < = 1: p-value = 0.0007, 3: p-value = 0.0013, 4: p-value = 0.04. Scale bars correspond to 10μm in all images.
(TIF)

S4 Fig (A) The *neur-LacZ* marked SOPs are regularly spaced in the heterozygous *MZ1369-GAL4* control. (B) Ubiquitous downregulation of *sns* using RNAi does not have any major impact on SOP spacing. (C) Ubiquitous downregulation of *hbs* and *sns* using RNAi results in a disrupted SOP pattern. SOPs are frequently found outside of the neurogenic rows of the presumptive wing margin and the spacing is irregular. (D) Similar phenotypes are displayed in the ubiquitous *sns* misexpression.
(TIF)

S5 Fig (A-P) High magnification images of projection views of IRM immunoreactivity in third instar larvae. Rst is shown in red (A, E, I and M), Hbs in green (B, F, J and N), Kirre in blue (C, G, K and O) and SNS in yellow (D, H, L and P). (A-D) Misexpression of *rst* using *neur-GAL4* leads to strong Rst staining of the entire SOP (A). Hbs (B) is found only around the SOPs. Staining of membranes not in contact to the SOPs is reduced. (C) Kirre staining is further enriched around the SOPs. (D) SNS is relocated and it is not specifically located at the adherens junction any more. Instead, the entire cell body is stained. (E-H) Misexpression of *kirre* using *neur-GAL4* leads to strong Rst staining (E) around the SOPs. Hbs (F) is found much stronger around or in the SOPs and is strongly reduced on the membranes not in contact with any SOPs. (G) Kirre staining is strongly found in all membranes of the SOPs. (H) SNS localization inside the SOPs is disrupted and the entire cell is immunopositive. (I-L) Misexpression of *hbs* via *neur-GAL4* leads to strong Rst stained membranes in contact with the SOPs (I). Membranes that are not in contact to SOPs show reduced staining. (J) Hbs staining can be found in the entire SOPs without profound apical basal polarity. (K) Kirre is similar to Rst enriched at the membranes in contact to the SOPs. Hardly any staining can be found in other membranes of the adhesive belt. (L) Cellular localization of SNS is only mildly affected by the *hbs* misexpression as the protein is not exclusively localized at the SOP membrane. Additionally, filopodial outgrowths are frequently observed in this genotype. (M-P) Overexpression of *sns* using *neur-GAL4* leads to strongly enriched staining of Rst (M), Hbs (N) and Kirre (O) around the SOPs. (P) SNS can be found along all membranes of the SOPs due to the overexpression. Similar to hbs overexpression are filopodial outgrowth frequently observed. (Q) In the adult *neur-GAL4* driven misexpression of *rst* significantly changes the spacing pattern and bristles cluster strongly and spacing ranges from 0 to >10 intervening cells. This is true for both chemosensory and mechanosensory bristles. (R) Similarly, *neur-GAL4* driven misexpression of *kirre* in the SOPs significantly changes the spacing pattern and bristles cluster strongly. Spacing ranges from 0 to >10 intervening cells. This is true for chemosensory and mechanosensory bristles. (S) *neur-GAL4* driven overexpression of *hbs* has only minor impact on bristle spacing (1 to 6 intervening cells) in the wing margin. (T) *neur-GAL4* driven overexpression of *sns* in the SOPs has only a very mild effect on bristle spacing (2 to 6 intervening cells). Quantitative analysis of the ventral spacing can be seen in (U). The distribution of *neur>GFP* differs significantly from *neur>rst* in the following spacing value: < = 1: p-value = 0.006, 3: p-value = 0.0002, 5: p-value = 0.0065, > = 6: p-value = 0.0004. *neur>kirre* differs significantly in the following spacing value: 3: p-value = 0.0002, 4: p-value = 0.0043, > = 6: p-value = <0.0001. *neur>hbs* differs significantly in the following spacing value: 2: p-value = 0.0056, 3: p-value = 0.0036, 4: p-value = 0.014. *neur>sns* differs significantly in the following spacing value: 3: p-value = 0.0057. Scale bars correspond to 10μm in all images.
(TIF)

S1 Table Bristle numbers on dorsal and ventral side of the anterior wing margin. The table shows the mean and standard error (SE) of the bristle counts from several genotypes used in this study. In the first column are the genotypes shown. The data columns are named as follows: dorsal triple row (dTR), middle triple row (mTr), dTR/mTr, ventral triple row recurved bristles (vTr r), ventral triple row slender bristles (vTr s) and vTr r/vTr s. Mean numbers shown are always half male half female as no sex differences were found. (T-test < 0.05) and with two asterisks for (T-test < 0.01) and three for (T-test <0.001).
(DOCX)
