# Supplementary figures and images for: The Cell Adhesion Molecules Roughest, Hibris, Kin of Irre and Sticks and Stones Are Required for Long Range Spacing of the Drosophila Wing Disc Sensory Sensilla
Source: PLoS One. 2015 Jun 8;10(6):e0128490. doi: 10.1371/journal.pone.0128490 (PMC4459997; doi:10.1371/journal.pone.0128490)

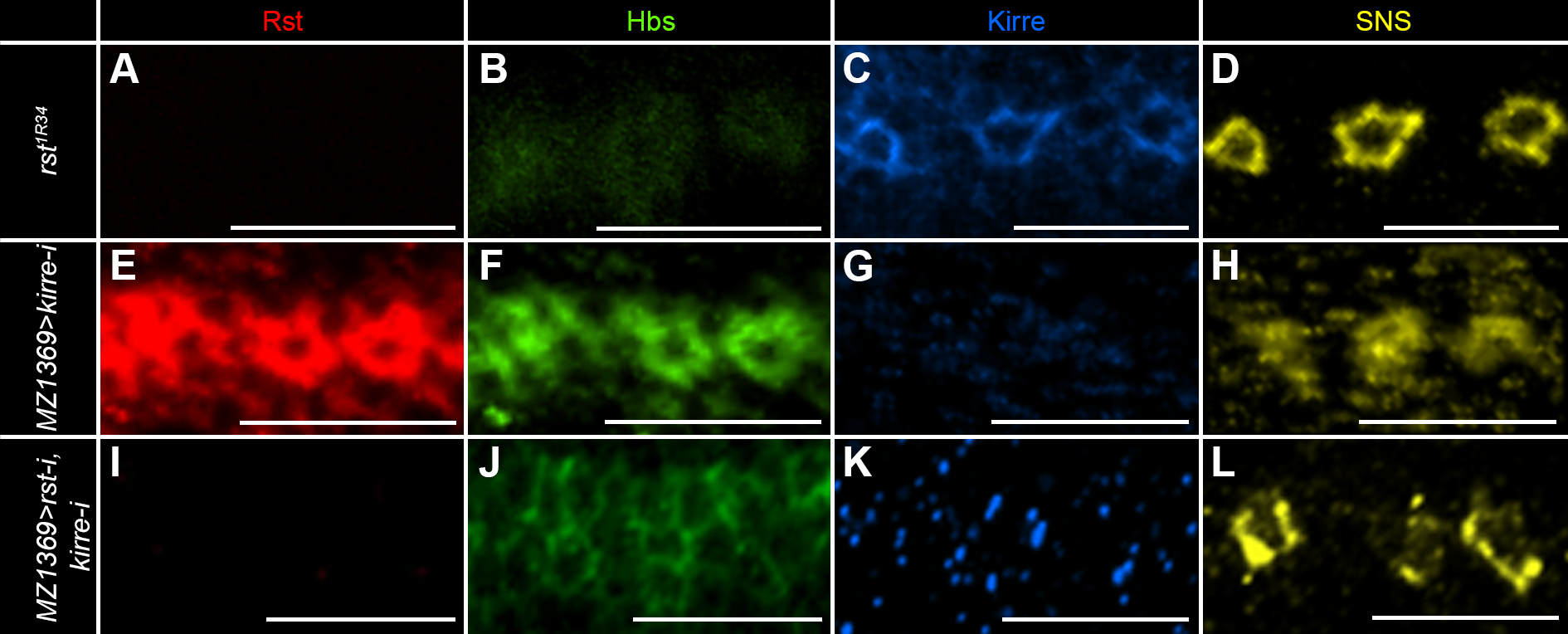

Supplement: S1 Fig — (A-L) High magnification images of projection views of IRM-protein immunoreactivity in late third instar larvae. Rst is shown in red (A, E, and I), Hbs in green (B, F and J), Kirre in blue (C, G and K) and SNS in yellow (D, H and L). (A-D) The rst allele rst 1R34 shows no detectable Rst staining. (B) Hbs staining is reduced, especially in the membranes surrounding the SOPs and the protein is mainly detected in SOP membranes. Kirre (C) and SNS (D) show no significant pattern change. (E-H) MZ1369-GAL4>UAS-kirre-RNAi shows no significant changes of the Rst (E) and Hbs pattern (F). Kirre immunoreactivity is hardly detectable (G) while SNS (H) is mildly reduced. (I-L) In the rst, kirre double RNAi hardly any Rst and Kirre (I, K) can be detected. Enrichment of Hbs (J) around SOPs is reduced. SNS (L) is not evenly distributed around the SOP membrane. Scale bars correspond to 10μm in all images. (TIF) [file pone.0128490.s001.tif]

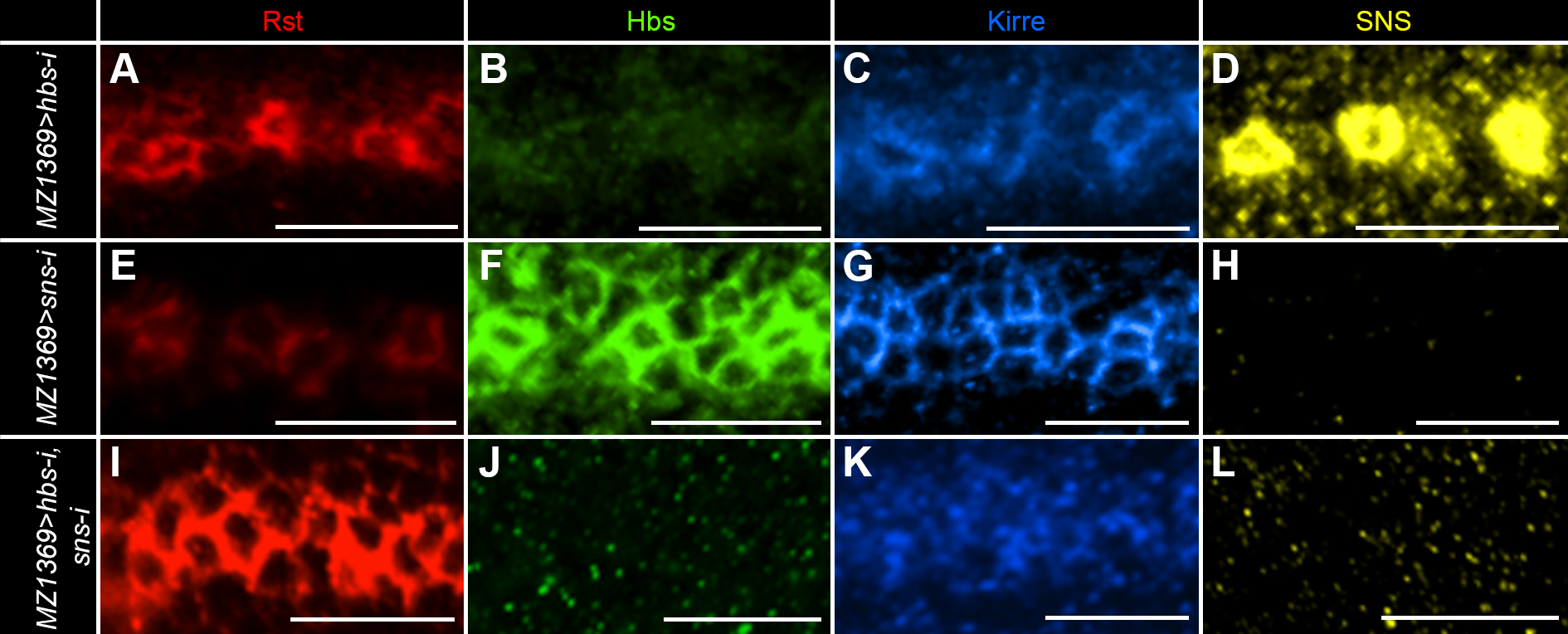

Supplement: S2 Fig — (A-L) High magnification images of projection views of IRM-protein immunoreactivity in late third instar larvae. Rst is shown in red (A, E and I), Hbs in green (B, F and N), Kirre in blue (C, G and K) and SNS in yellow (D, H and L). (A-D) Global hbs-RNAi using MZ1369-GAL4 reduces the staining for Rst (A) and Kirre (C) in all membranes that are not in contact to the SOPs (B) Hbs immunoreactivity is reduced and no clear membrane localization is detectable. (D) SNS immunoreactivity is mildly stronger. (E-H) MZ1369-GAL4>UAS-sns-RNAi shows mildly reduced Rst staining. Hbs (F) and Kirre (K) immunoreactivity is unchanged. SNS (H) is not detectable. (I-L) In the double RNAi MZ1369>hbs-RNAi, SNS-RNAi the two adhesive belts with Rst (I) and Kirre (K) are visible, but no obvious SOPs. Hbs (J) and SNS (L) are not detectable. Scale bars correspond to 10μm in all images. (TIF) [file pone.0128490.s002.tif]

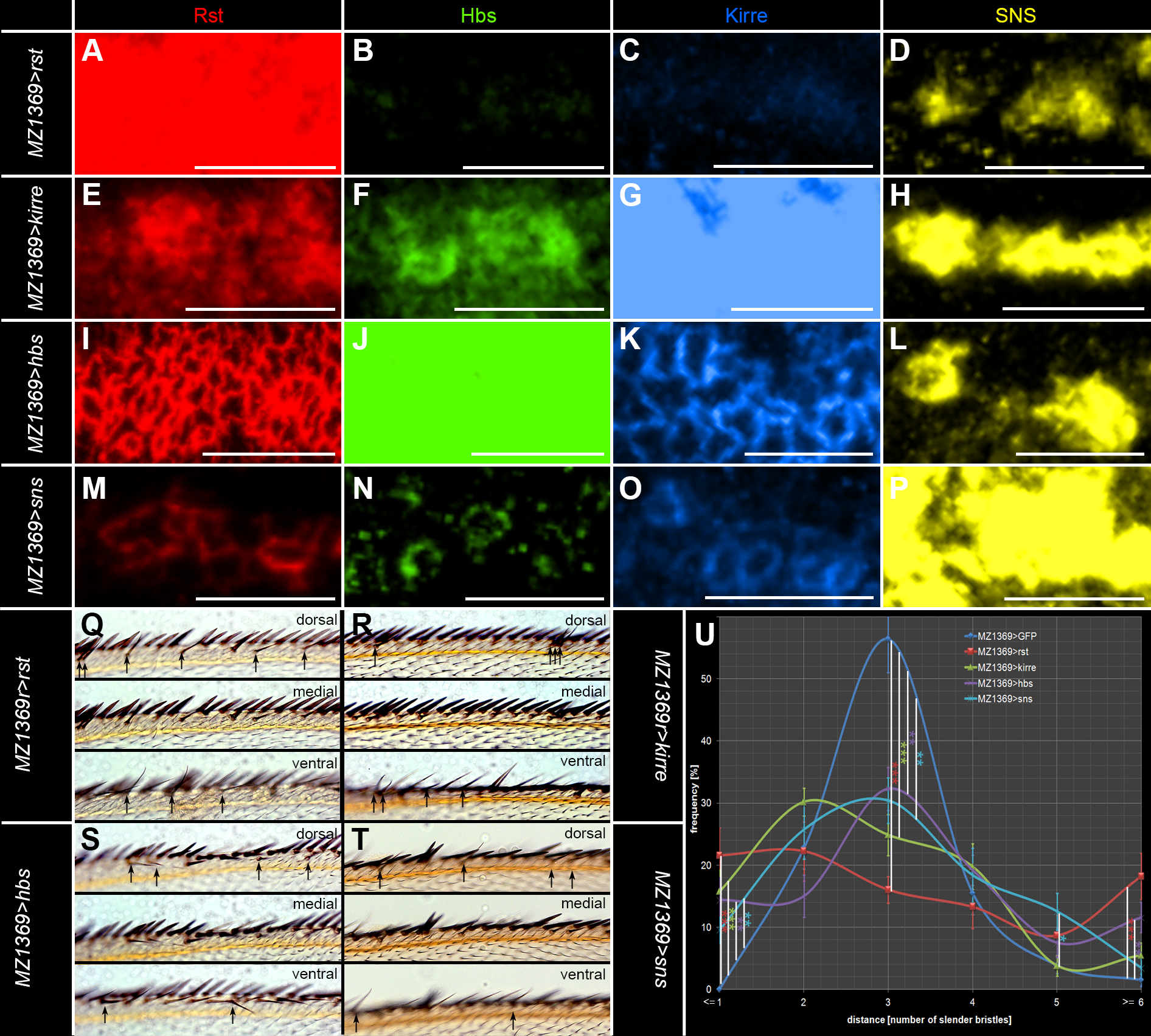

Supplement: S3 Fig — (A-P) High magnification images of projection views of IRM immunoreactivity in third instar larvae. Rst is shown in red (A, E, I and M), Hbs in green (B, F, J and N), Kirre in blue (C, G, K and O) and SNS in yellow (D, H, L and P). (A-D) Misexpression of rst using MZ1369-GAL4 leads to ubiquitous Rst staining (A). Hbs (B) and Kirre (C) are significantly reduced and enrichment around SOPs is not visible anymore. SNS (D) staining is unaffected in strength, but the localization is not limited to the apical contact zone of the SOPs. Instead it is found in the entire cell. (E-H) Misexpression of kirre via MZ1369-GAL4 leads to wider and less membrane specific stripes of Rst (E) and Hbs (F) staining. Kirre can be ubiquitously detected (G). SNS (H) staining is strong on all membranes in contact with Kirre membranes. (I-L) Misexpression of hbs using MZ1369-GAL4 leads to a significant enlargement of Rst (I) and Kirre (L) immunoreactivity positive areas. All membranes of the adhesive belt show increased immunoreactivity and specific enrichment around the SOPs is lost. Hbs (K) is ubiquitously located in all membranes. SNS (L) staining shows that the SOPs have lost their regular pattern already in this developmental stage. (M-P) Ubiquitous misexpression of sns via MZ1369-GAL4 leads to reduced staining of Rst (M), Hbs (N) and Kirre (O). SNS (P) staining shows patched expression. (Q) In the adult MZ1369-GAL4 misexpression of rst has a strong impact on the spacing of recurved bristles and spacing ranges from 0 to 13 intervening cells. Clustered recurved bristles are frequently observed and also long areas without any chemosensory bristles are seen. (R) MZ1369-GAL4 driven kirre misexpression has a strong impact on the spacing of recurved bristles with spacing ranging from 0 to 7. Clustered recurved bristles are frequently observed similarly are also long are areas without any chemosensory bristles seen. (S) MZ1369-GAL4 misexpression of hbs has a strong impact on the spacing of recurv [file pone.0128490.s003.tif]

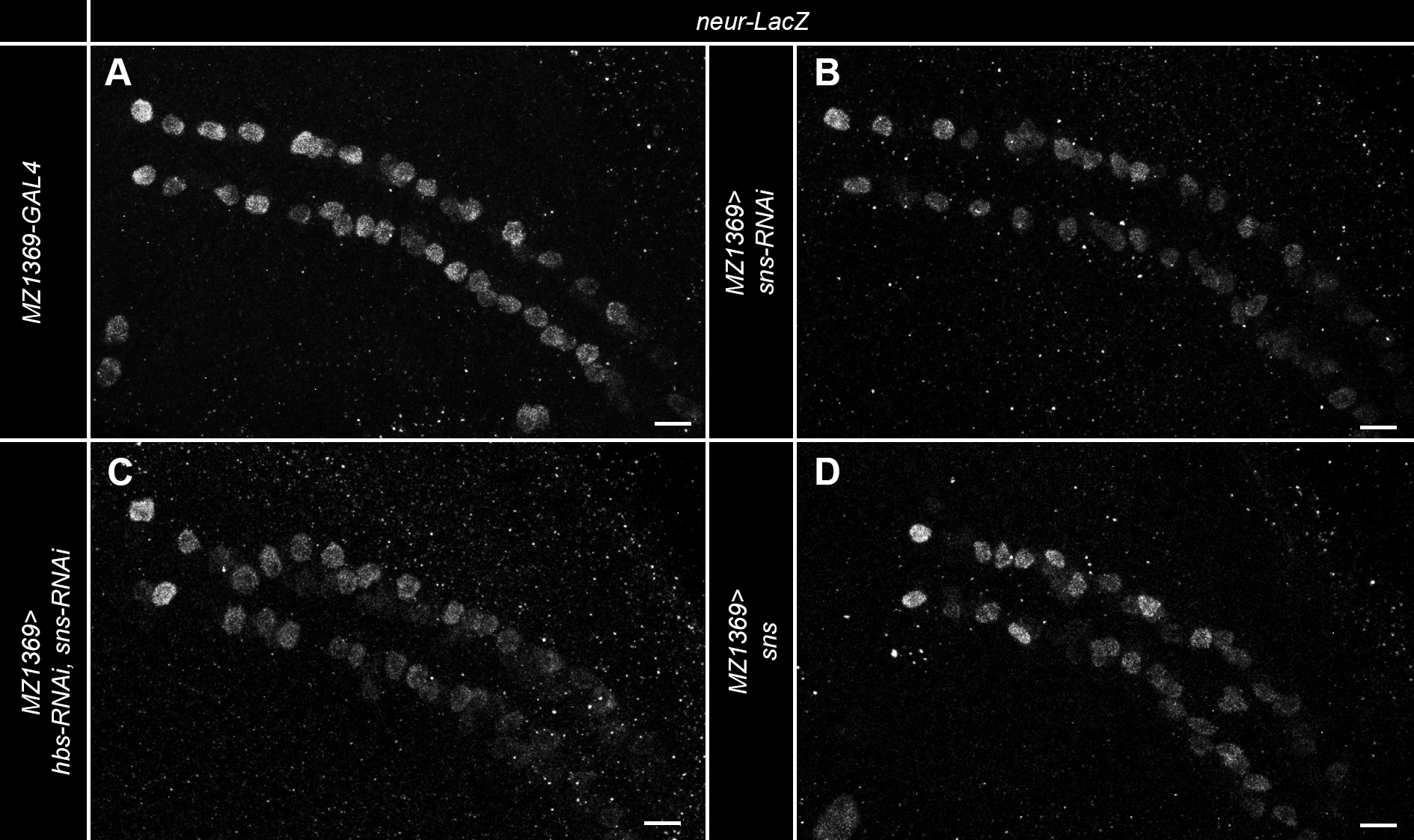

Supplement: S4 Fig — (A) The neur-LacZ marked SOPs are regularly spaced in the heterozygous MZ1369-GAL4 control. (B) Ubiquitous downregulation of sns using RNAi does not have any major impact on SOP spacing. (C) Ubiquitous downregulation of hbs and sns using RNAi results in a disrupted SOP pattern. SOPs are frequently found outside of the neurogenic rows of the presumptive wing margin and the spacing is irregular. (D) Similar phenotypes are displayed in the ubiquitous sns misexpression. (TIF) [file pone.0128490.s004.tif]

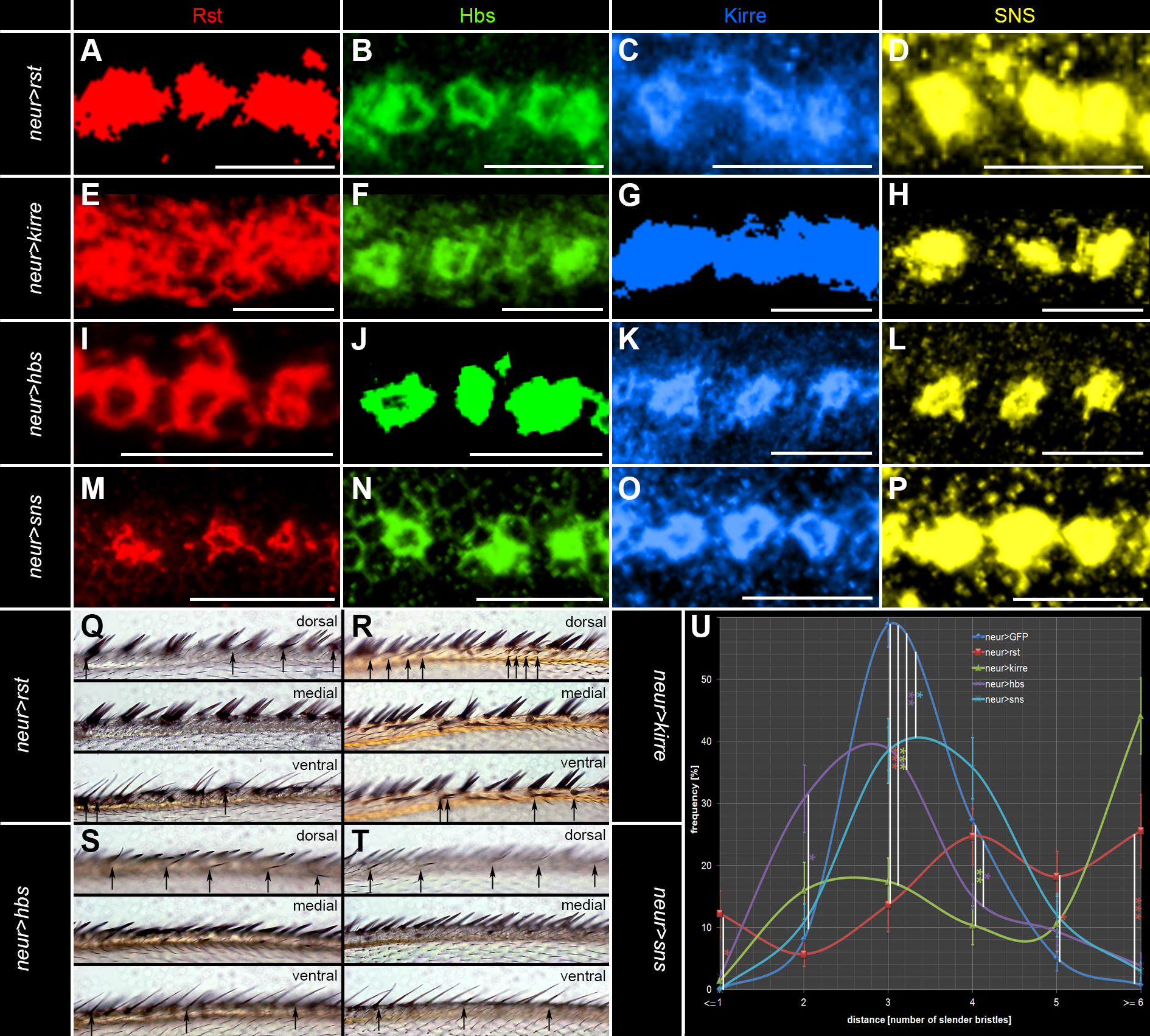

Supplement: S5 Fig — (A-P) High magnification images of projection views of IRM immunoreactivity in third instar larvae. Rst is shown in red (A, E, I and M), Hbs in green (B, F, J and N), Kirre in blue (C, G, K and O) and SNS in yellow (D, H, L and P). (A-D) Misexpression of rst using neur-GAL4 leads to strong Rst staining of the entire SOP (A). Hbs (B) is found only around the SOPs. Staining of membranes not in contact to the SOPs is reduced. (C) Kirre staining is further enriched around the SOPs. (D) SNS is relocated and it is not specifically located at the adherens junction any more. Instead, the entire cell body is stained. (E-H) Misexpression of kirre using neur-GAL4 leads to strong Rst staining (E) around the SOPs. Hbs (F) is found much stronger around or in the SOPs and is strongly reduced on the membranes not in contact with any SOPs. (G) Kirre staining is strongly found in all membranes of the SOPs. (H) SNS localization inside the SOPs is disrupted and the entire cell is immunopositive. (I-L) Misexpression of hbs via neur-GAL4 leads to strong Rst stained membranes in contact with the SOPs (I). Membranes that are not in contact to SOPs show reduced staining. (J) Hbs staining can be found in the entire SOPs without profound apical basal polarity. (K) Kirre is similar to Rst enriched at the membranes in contact to the SOPs. Hardly any staining can be found in other membranes of the adhesive belt. (L) Cellular localization of SNS is only mildly affected by the hbs misexpression as the protein is not exclusively localized at the SOP membrane. Additionally, filopodial outgrowths are frequently observed in this genotype. (M-P) Overexpression of sns using neur-GAL4 leads to strongly enriched staining of Rst (M), Hbs (N) and Kirre (O) around the SOPs. (P) SNS can be found along all membranes of the SOPs due to the overexpression. Similar to hbs overexpression are filopodial outgrowth frequently observed. (Q) In the adult neur-GAL4 driven misexpression of rst significantly changes the s [file pone.0128490.s005.tif]
