# Supplementary material for: The Cell Adhesion Molecules Roughest, Hibris, Kin of Irre and Sticks and Stones Are Required for Long Range Spacing of the Drosophila Wing Disc Sensory Sensilla
Source: PLoS One. 2015 Jun 8;10(6):e0128490. doi: 10.1371/journal.pone.0128490 (PMC4459997; doi:10.1371/journal.pone.0128490)
Supplement: S1 Table — The table shows the mean and standard error (SE) of the bristle counts from several genotypes used in this study. In the first column are the genotypes shown. The data columns are named as follows: dorsal triple row (dTR), middle triple row (mTr), dTR/mTr, ventral triple row recurved bristles (vTr r), ventral triple row slender bristles (vTr s) and vTr r/vTr s. Mean numbers shown are always half male half female as no sex differences were found. (T-test < 0.05) and with two asterisks for (T-test < 0.01) and three for (T-test <0.001). (DOCX) [file pone.0128490.s006.docx]

| **genotype** |  | **dTr** | **mTr** | **mTr/dTr** | **vTr r** | **vTr s** | **vTr s/vTr r** |
| --- | --- | --- | --- | --- | --- | --- | --- |
| ***wtb*** | **Mean** | **18.1** | **83.6** | **4.6** | **12.4** | **45.5** | **3.7** |
| (n=10) | SE | ± 0.4 | ± 1.3 | ± 0.1 | ± 0.3 | ± 1.2 | ± 0.1 |
| ***neur*-GAL4>UAS-*mCD8-*GFP** | **Mean** | **18.5** | **88.1** | **4.8** | **14.1** | **48.0** | **3.4** |
| (n=10) | SE | ± 0.4 | ± 1.5 | ± 0.1 | ± 0.5 | ± 1.0 | ± 0.1 |
| ***neur-GAL4*>*UAS-rst-RNAi*** | **Mean** | **20.1 *** | **91.9 *** | **4.6** | **14.9** | **53.5 **** | **3.6** |
| (n=10) | SE | ± 0.3 | ± 1.3 | ± 0.1 | ± 0.4 | ± 1.2 | ± 0.1 |
| ***neur-GAL4*>*UAS-rst*** | **Mean** | **11.5 **** | **92.7** | **8.1 **** | **9.9 **** | **46.0** | **4.9 *** |
| (n=10) | SE | ± 0.3 | ± 2.6 | ± 0.4 | ± 0.6 | ± 2.6 | ± 0.6 |
| ***neur-GAL4*>*UAS-kirre-RNAi*** | **Mean** | **19.3** | **87.8** | **4.6** | **13.8** | **51.1 *** | **3.7 *** |
| (n=10) | SE | ± 0.3 | ± 1.9 | ± 0.1 | ± 0.3 | ± 0.7 | ± 0.1 |
| ***neur-GAL4*>*UAS-kirre*** | **Mean** | **9.9 **** | **86.8** | **9.0 **** | **7.9 **** | **43.4 *** | **5.8 **** |
| (n=10) | SE | ± 0.6 | ± 2.4 | ± 0.6 | ± 0.6 | ± 0.9 | ± 0.5 |
| ***neur-GAL4*>*UAS-hbs-RNAi*** | **Mean** | **19.5 *** | **92.5 *** | **4.8** | **14.2** | **50.9 *** | **3.6** |
| (n=10) | SE | ± 0.3 | ± 1.6 | ± 0.1 | ± 0.4 | ± 0.6 | ± 0.1 |
| ***neur-GAL4*>*UAS-hbs*** | **Mean** | **20.7 *** | **90.3** | **4.4 *** | **15.6 *** | **49.9** | **3.2** |
| (n=10) | SE | ± 0.8 | ± 2.3 | ± 0.1 | ± 0.6 | ± 1.4 | ± 0.1 |
| ***neur-GAL4*>*UAS-sns-RNAi*** | **Mean** | **18.4** | **83.3** | **4.57** | **14.1** | **49.3** | **3.5** |
| (n=10) | SE | ± 0.6 | ± 2.4 | ± 0.2 | ± 0.4 | ± 0.6 | ± 0.1 |
| ***neur-GAL4*>*UAS-sns*** | **Mean** | **15.5 **** | **85.6** | **5.6 **** | **12.5 **** | **46.7** | **3.8 *** |
| (n=10) | SE | ± 0.5 | ± 1.0 | ± 0.2 | ± 0.4 | ± 0.9 | 0.1 |
